# Failure wear mechanism and blade width effect of TBM center cutter rotation in water-bearing weakly cemented sandstone

**DOI:** 10.1371/journal.pone.0354108

**Published:** 2026-09-25

**Authors:** Qing Yang, Shuchen Li, Xiuwei Wang

**Affiliations:** 1 School of Mechanics and Civil Engineering, China University of Mining and Technology, Xuzhou, China; 2 School of Intelligent Construction and Hydraulic Engineering, Chuzhou University, Chuzhou, China; 3 Anhui Provincial Highway Construction Low Carbon Materials Engineering Research Center, Chuzhou University, Chuzhou, China; 4 Geotechnical Engineering Department, Chuzhou Architectural Survey and Design Institute Co., Ltd, Chuzhou, China; 5 School of Future Technology, Shandong University, Jinan, China; The University of Hong Kong, HONG KONG

## Abstract

Weakly cemented sandstone (WCS) in western China is rich in clay minerals. Water‑induced softening tends to induce rotation failure of TBM central hobs, which further causes eccentric tip damage and edge chipping and remarkably reduces tunnelling efficiency. Aiming at the poorly understood sliding‑contact mechanism of central hobs under rotation‑failure conditions in WCS strata, steel‑needle scratching tests combined with XRD and SEM micro‑characterization were carried out to investigate the apparent morphological evolution of hob tips, and a corresponding empirical correlation was fitted. The results show that water saturation may promote softening of clay cement, trigger the generation of rock slag and give rise to secondary interfacial abrasion. Measured data from WCS scratching tests indicate that the relative apparent‑diameter variation of the 0.4 mm needle for water‑saturated specimens is 1.53 times that of the 1.0 mm needle, and the geometric variation of the 0.4 mm needle increases by 45%‑57% compared with dry conditions. With correction terms of equivalent blade width and water content incorporated, the empirical correlation reproduces tip‑morphology variation trends that are generally consistent with field observations of domestic TBM projects. Based on scaled steel‑needle tests, this study reveals the corresponding evolution law of tip‑contact responses for TBM central hobs under rotation‑failure conditions in Cretaceous WCS strata, and can provide experimental basis and theoretical references for hob structural design, cutter‑head layout and tunnelling‑parameter optimization for such strata. This mechanistic analysis relies on quasi‑static scratching tests and focuses on sliding‑contact behaviours at the tool‑rock interface. Its engineering application should be comprehensively evaluated combined with in‑situ dynamic tunnelling conditions.

## Introduction

As the core equipment in underground engineering, the full-face tunnel boring machine (TBM) is widely used in major projects such as the Western Railway and water conservancy [1]. However, its problem with hob wear is particularly pronounced in weakly cemented sandstone formations [2]. Due to weak particle cementation, high content of quartz and feldspar, and clay minerals such as illite and montmorillonite in the Cretaceous Jurassic strata in the west, the cementation strength is significantly reduced after encountering water. The rock debris generated by rock breaking easily adheres to the hob, forming a “mud cake”, and the phenomenon of rock debris argillization and sandification will aggravate the hob’s secondary wear. The wear mechanism is essentially different from that of hard rock [2,3]; The wear law of the central cutter area is more complex due to repeated crushing of rock slag, and the difference between the central cutter area and other cutters is particularly significant [4,5]. The central cutter is located at the geometric center of the cutter head, with a very small radius of revolution and low linear velocity. The weakly cemented sandstone (WCS) of the Cretaceous system is rich in quartz, feldspar, and hydrophilic clay minerals. Under these geological conditions, the TBM center blade is prone to self rotation blockage and even self rotation failure. It primarily completes the initial crushing and compaction of the rock mass in the central area through sliding grinding, thereby providing the basic conditions for the stable cutting by the surrounding cutters. The wear state of the center cutter directly determines the formation quality of the free face and the subsequent rock breaking efficiency, so it is of great engineering significance to explore the wear problem of the center cutter.

For the wear of TBM hobs, many scholars have carried out a lot of research around the edge structure: Liu Ying Chao [6] compared the rock breaking characteristics of sharp edge, arc edge, and tooth edge hobs through similar material tests, and found that the sharp blade is prone to wear due to stress concentration. Based on this, the new tooth edge wedge cutter can significantly reduce the wear and improve the rock breaking efficiency; Xia et al. [7–9] found in the rock breaking test with the V-shaped edge that the tool forms a curved surface or planar structure with a specific tip width after passivation, and the changes of its contour shape, edge radius and other parameters will affect the load of the hob. The edge shape, as the key factor restricting the contact behavior between the hob and the rock mass [3], has a particularly pronounced impact on tool wear at specific positions, such as the center tool.

Starting from the plastic removal mechanism of abrasive wear, Yang Yan Dong et al. [10] pointed out that hard particles (such as quartz) in such weakly cemented sandstone are easy to press into the surface of the cutter ring under the vertical force of the hob, forming a furrow effect and accelerating the cutting of the tool material. At the same time, Jiang Yu Sheng [11] confirmed that the process of the formation of the groove in the rock carved by the steel needle is consistent with the rock breaking process accompanied by torsional shear under the vertical thrust of the TBM. The furrow effect is positively correlated with the metal wear rate [12], providing a theoretical basis for the analysis and simulation testing of the hob wear mechanism. Yao et al. [13] confirmed through abrasion test that the abrasion characteristics of WCS are significantly controlled by water content, and water saturation will weaken the rock cementation structure and change the tool wear form; Another study [14] shows that formation argillization effect will further aggravate hob wear, which is enough to show that water rock coupling is the core influencing factor of tool wear in this kind of formation.

Tool wear at different positions on the TBM cutter head is directly affected by the installation radius. Zhao Zhanxin [15] made it clear that the larger the tool’s installation radius, the more severe the wear, except for the center tool. The actual measurement of a TBM project in Xinjiang [16] further verified that the wear of the center cutter was mainly due to lateral slip. The abnormal wear rate (eccentric wear and edge collapse) of the center cutter in TBM projects such as the Gaoligongshan tunnel [17] of the Darui railway and the Lanzhou Water Source Area bid 2 [18] reached 77.8% and 90.6%, respectively. In addition, due to the minimum installation radius, the center cutter also faces unique wear incentives: the rock impact on the blade is more concentrated, which is prone to plastic deformation; Chen Yu Kun et al. [19,20] also showed that the radial wear was closely related to the radius and speed of the installation position, which further highlighted the importance of the installation position for wear analysis; Zhang Zhao Huang et al. [21] pointed out that the rock breaking force of disc hobs with uneven wear increases with the increase of wear.

There are obvious shortcomings in the current related research. Most research focuses on the conventional rolling wear of tools in hard rock formations. There is little research on the wear mechanism caused by the weakening cementation of WCS in water, and on the argillization and adhesion of rock slag, and there is also a lack of specialized analysis of pure sliding wear under the condition of central tool rotation failure. In terms of test methods, traditional large-scale cutting test equipment is expensive and offers limited adjustability of working conditions, making it difficult to conduct comparative tests across multiple parameter groups, such as moisture content and blade width. With the advantages of low cost and high controllability, simplified indoor tests such as steel-needle engraving and small-scale abrasion have been widely used to study rock abrasion and tool wear [11].

It should be noted that indoor scaled model testing is not inherently equivalent to the physical equivalence of prototype engineering. The size effect caused by scaling replacement can lead to differences in micro and macro responses, and extrapolating indoor test results to engineering prototypes requires careful evaluation [22]; The multi field coupling damage composed of water, temperature, and excavation cycle disturbance controls the mechanical behavior of rock cutting contact, and quasi-static indoor loading conditions are difficult to fully reproduce the complex service environment on site [23]. Further constitutive theory studies have shown that soil constitutive anisotropy, bond degradation, and loading modes significantly constrain the transformation of indoor empirical relationships to prototype conditions, and the relevant relationships obtained from experiments must clarify their applicable boundaries [24]. Based on the existing research, although this type of simplified scratching test can reproduce core mechanisms such as stress concentration at the rock cutting interface, rock water softening, and sliding abrasion, it is limited by quasi-static unidirectional loading conditions and cannot reproduce real working conditions such as TBM rolling and sliding rotation, transient impact, and rock debris discharge. When promoting the test results to engineering prototypes, full attention should be paid to their equivalent boundaries. Therefore, when conducting experimental analysis, it is necessary to distinguish between the reproducible physical mechanisms of the experimental system and the engineering scale characteristics that cannot be simulated.

However, existing experiments have not taken into account the special service conditions of TBM center blade rotation failure and continuous sliding grinding, nor have they explored the evolution of needle tip morphology under the coupling effect of blade width and moisture content. In view of this, this article focuses on the WCS formation and simulates the center blade sliding grinding process of TBM self rotation failure using a scaled steel needle cutting test. The effects of blade width and moisture content on frictional resistance, cutting depth, and apparent diameter changes of the needle tip are analyzed, and a preliminary indoor empirical correlation relationship for center blade wear considering blade width and moisture content correction is constructed.

### Engineering background and wear characteristics analysis

Due to weak particle cementation, high contents of quartz and feldspar, and hydrophilic clay minerals, the cementation strength of the WCS formation decreases significantly upon exposure to water, and the rock mass is prone to argillization and adhesion, which is one of the most prominent formation types of TBM hob wear. In order to clarify the wear characteristics and universality of the center cutter in this kind of formation, the service conditions of the center cutter in several TBM projects crossing WCS or similar soft formation in China are counted, as shown in Table 1.

**Table 1:** Statistics of the number and wear of some TBM center cutter hobs in China.

Serial Number	Quantity/piece; Specification/in						Important conclusions or facts	Application Engineering Name
	Center cutter		Positive cutter		Edge cutter			
1	4 (pairs)	17	52	19	8	19	After changing the central cutter, reduce the speed and advance speed, and recover the tunneling parameters according to the characteristics of different surrounding rocks after 50 ~ 60 cm running in.	TBM of the West Qinling Extra Long Tunnel on the Lanyu Line [15].
2	4 (pairs)	17	35	20	10	20	The wear of the cutting tools is highly correlated with the erosion properties of the rocks. The higher the erosion index of the rocks, the greater the wear per meter of the cutting tools.	TBM of a tunnel in Northeast China [25].
3	4 (pairs)	17	34	19	10	19	Average diameter wear rate (mm/m^3^): Center cutter 0.1416; Positive cutter 0.0776; Edge cutter 0.3071.	A TBM project in Xinjiang [16].
4	6	17	62	20	5	20	161 double-edged center knives were replaced, and 101 were replaced due to wear, chipping, or other damage, accounting for 62.7%.	TBM of Hanji Wei Qinling Tunnel [26].
5	6	17	21	19	10	19	The total excavation length was 3,239 meters, with 32 abnormal cutter replacements for the central cutter, 23 instances of uneven wear, and 6 cases of cutter ring loosening.	Lanzhou Water Source Area Section 2 [18].
6	8	17	31	19	10	19	The regression analysis of 63 sets of data shows the fitting relationship between the equivalent quartz content and uniaxial compressive strength of rocks and their impact on abrasion resistance.	Northern Xinjiang Water Supply TBM [27].
7	4 (pairs)	17	42	19	12	19	9 center rolling cutters were replaced, 5 were unevenly worn, and 2 cutter rings were broken. The average rock breaking volume wear rate of the central rolling cutter is 0.044 mm/m^3^, the positive rolling cutter is 0.022 mm/m^3^, and the edge rolling cutter is 0.093 mm/m^3^	Dali-ruili railway Gaoligongshan Tunnel [17].
8	4 (pairs)	17	31	20	12	20	The running radius of the center blade is small, resulting in severe lateral sliding and wear of the rolling cutter. The larger the installation radius, the smaller the lateral sliding and wear of the rolling cutter.	Introduce the TBM of Han Ji Wei Lingnan [28].
9	6 (pairs)	17	24	19	12	19	The center cutter has a deviation of 94.12% on the left line and 80% on the right line. The center cutter is mostly worn due to the formation of mud cakes.	Shenzhen Metro Line 13 [29].

Note: 17 inches (diameter 432 mm, blade width 8–10 mm); 19 inches (diameter 483 mm, blade width 12–15 mm); 20 inches (diameter 508 mm, blade width 15–18 mm).

It can be seen from Table 1 that eccentric wear and abnormal wear (edge collapse and tool ring fracture) are common in TBM center cutters in mud cake formation, and the eccentric wear rate of center cutters in some projects is as high as 94.12%. The central cutter is usually arranged in pairs, with a very small installation radius and low rotational linear speed, which makes it prone to rotation failure; the wear mode is mainly lateral sliding wear. The unit rock breaking volume wear rate of the center cutter is significantly higher than that of the front cutter. In a TBM project [16] in Xinjiang and the Gaoligongshan tunnel [17] project of the Darui railway, the unit rock-breaking volume wear rate of the center cutter is about twice that of the front cutter. Moreover, it is highly correlated with rock abrasiveness and water content. Mud cake adhesion and rock slag argillization are the main factors driving partial wear. Abnormal wear of the center cutter will not only lead to a high replacement frequency of the cutter and a sharp increase in construction costs, but also damage the empty face of the tunnel, reduce the rock-breaking efficiency of subsequent cutters, and even pose safety risks such as cutter head sticking. The contact area of the center cutter is smaller, the stress concentration effect is stronger, and it is more likely to intensify interfacial shear and local abrasion, which is one of the key drivers of the prominent problems of center-cutter wear and eccentric wear. In WCS formation, the wear mechanism of the center cutter is different from that of the conventional rolling wear in hard rock formation. The existing hob wear theory, based on hard rock conditions, is difficult to apply directly, so it is urgent to conduct targeted research into the sliding wear characteristics of the center cutter under rotational failure.

### Test scheme design

#### Marking test and data processing process.

**Fig 1:**
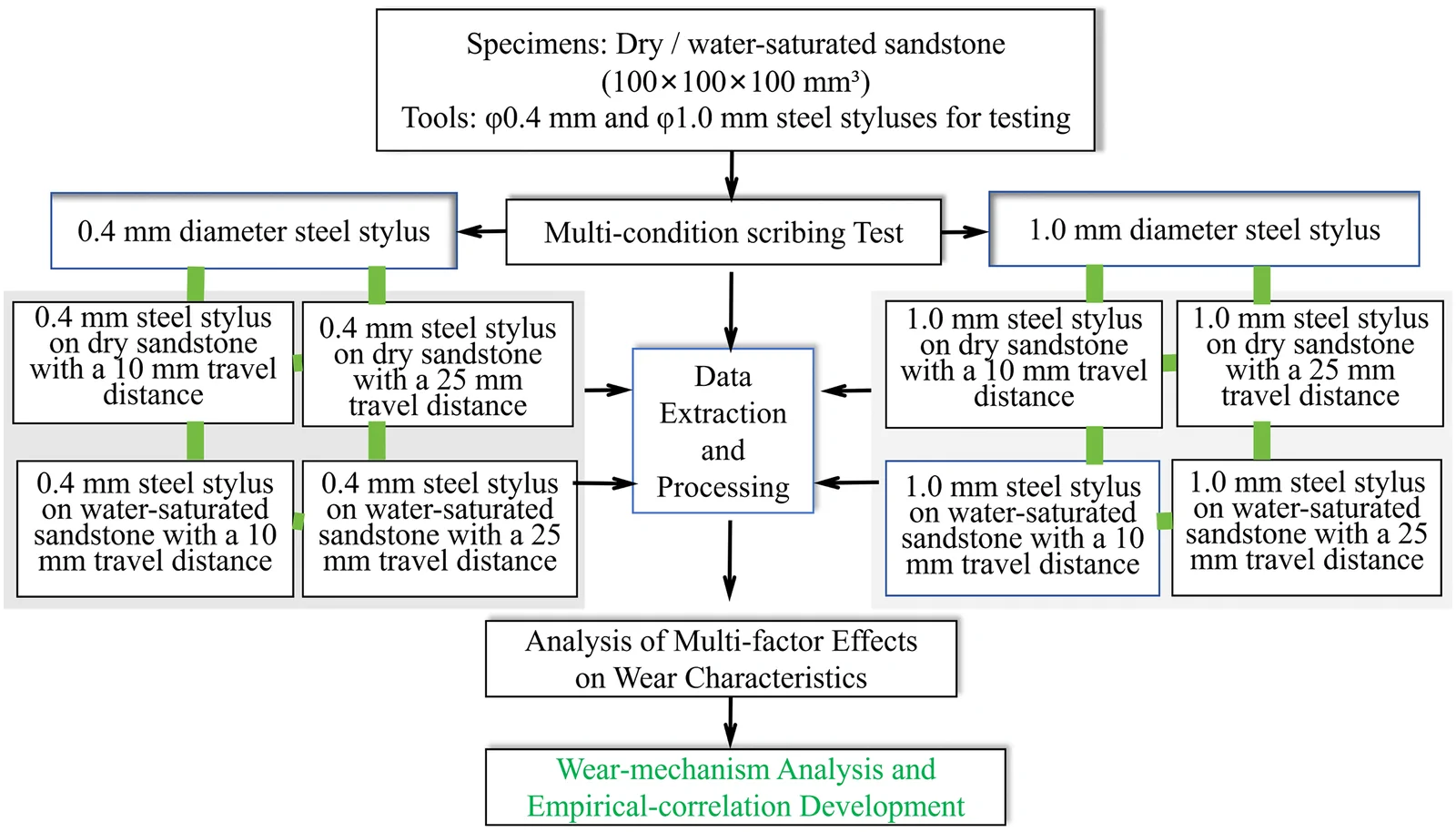
Workflow of steel-needle scribing test and data analysis.

This article carries out steel needle engraving tests, data analysis, and empirical correlation analysis in sequence. Using steel needle blade width, moisture content, and engraving stroke as variables, measure characteristic parameters such as friction force, engraving depth, and changes in the apparent diameter of the steel needle tip, analyze the influence of each variable on the evolution characteristics of the needle tip morphology, and ultimately construct an empirical correlation relationship for wear evolution. The test and data analysis process is shown in Fig 1.

The scribing distance for each segment was set to 25 mm. Four consecutive scribing cycles were performed under each test condition. The feed rate was 10 mm/min with a sampling frequency of 1 Hz, and approximately 150 measuring points were collected for each 25-mm run. Characteristic parameters in the steady-state stage were extracted for every scribing cycle. The complete set of scribing sequences for one steel-needle was regarded as one observation sample. Characteristic indices of each cycle were calculated for comparative analysis among different working conditions.

### Test device

**Fig 2:**
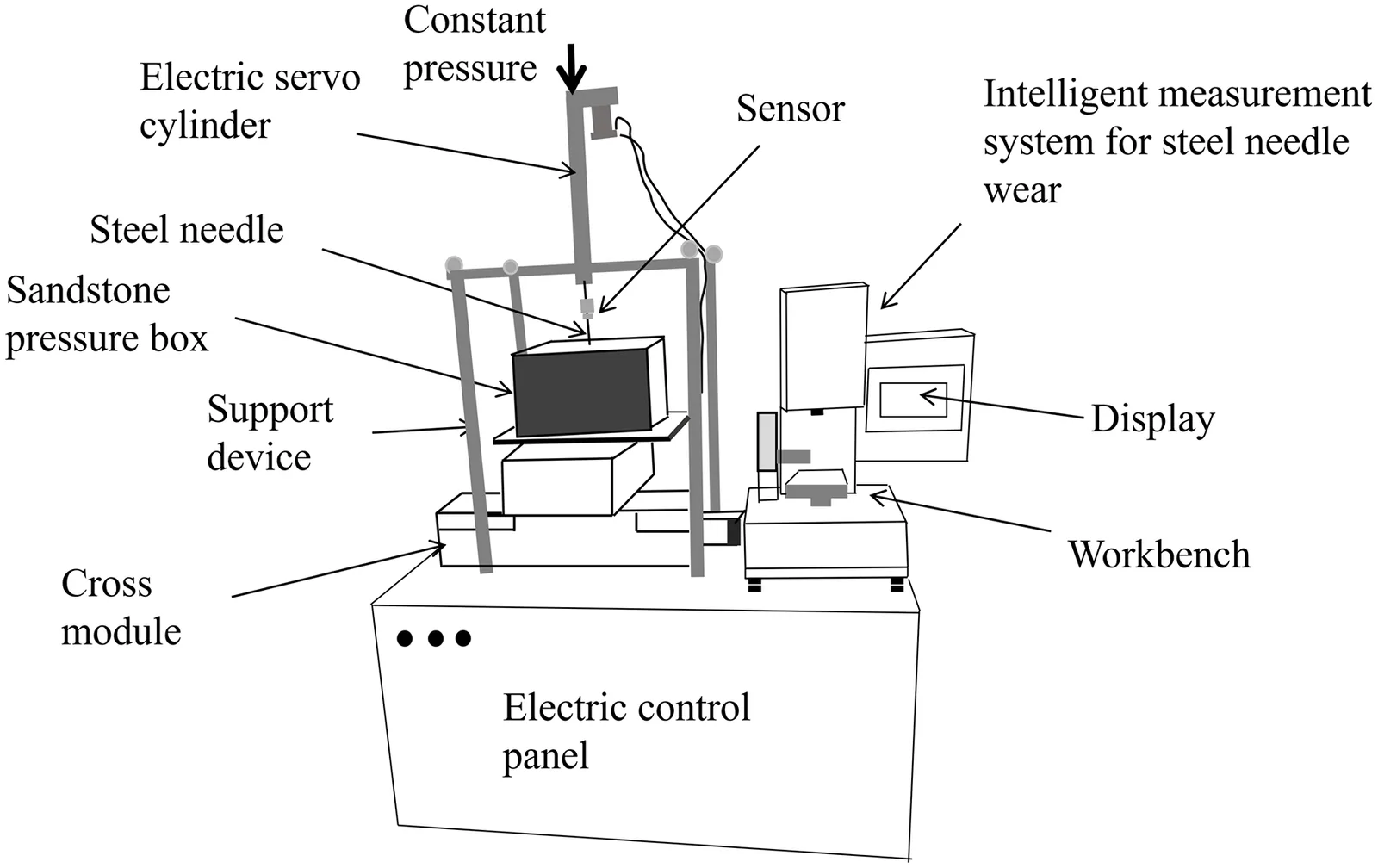
Schematic diagram of the YMC-1 intelligent test apparatus.

The YMC-1, a new intelligent Cerchar rock abrasion test system, was used for the test. The test equipment consists of a rock friction tester, an intelligent measuring device for steel needle wear, and a control system, as shown in Fig 2. The rock friction tester is composed of a sample clamp, a servo motor lateral displacement adjustment module, a servo motor vertical displacement adjustment module, a manual/automatic integrated displacement axial adjustment module, a displacement indicator scale, an alloy steel needle, and a steel needle holder. Double servo motors and program control are used to ensure the stable control of the relative movement (speed and displacement) between the rock sample and the steel needle. The steel needle wear intelligent measurement system includes a Gigabit Ethernet industrial array camera, a customized lens light source, a steel needle adaptive positioning clamping module, a fixed angle automatic rotation module, and an image information acquisition and processing system. The measurement control system comprises a fully digital closed-loop controller, a sensor, data-processing software, and a computer processing system. The sensor can measure the friction between the steel needle and the rock sample in real time. The intelligent software system can complete intelligent measurement and data processing. The horizontal force measurement accuracy is ± 0.003 mm, and the acquisition frequency for measuring friction is not less than 1000 Hz.

The constant vertical pressure of 70 N is set in the test, and the core basis is the actual stress accounting results of TBM construction [30]. Referring to the test report on rock abrasion resistance of Qingdao metro tunnel, and based on the maximum thrust of dsuc double shield TBM, the equivalent surface pressure of the cutter head is about 0.77 MPa (about 7.7 KGF); Considering the operation stability of the equipment, the load should be controlled within 90% of the service limit. Based on this, the reasonable equivalent surface pressure is calculated to be about 7.0 kgf, and 70 N is used as the equivalent design basis for the normal load in the steel needle scoring test.

Japanese tool steel SKD11 with a hardness of HRC58–60 is used as the special steel needle for the abrasion test of the steel needle. Its manufacturing process is rough turning → heat treatment → fine turning → fine grinding. The overall dimensions of the steel needle are 8 mm in diameter, 80 mm in length, and a 60°cone angle. There were two groups of needle tip diameters of 0.4 mm and 1 mm, with 4 needles in each group. The horizontal propulsion speed was set at 10 mm/min, and the test set two kinds of scoring strokes. The scoring stroke of 10 mm was used to analyze the instantaneous characteristics of mechanical response, and the scoring stroke of 25 mm was used to verify the scoring wear characteristics; The horizontal friction is collected in real time by a high-precision force sensor with a range of 0–200 N and a precision of 0.1 N. the depth curve and vertical force displacement curve are recorded by a synchronous laser displacement meter with a range of 0–5 mm and a precision of 0.01 mm.

### Sample preparation

The WCS of a mine in the west is selected as the test object, and its lithology is consistent with that of the engineering site. In the drying group, the samples were dried in a 105 °C oven for 24 hours to ensure that the moisture content was ≤ 0.5%. The saturated group used the vacuum pumping method (vacuum degree 0.09 MPa) to saturate the dried sample for 24 hours, and the final water absorption reached 6.62%, simulating the field groundwater environment. A WDW-300 universal testing machine (accuracy ± 0.5%) was used to determine the physical and mechanical parameters of the samples. For each group of three parallel samples, the results were averaged. The specific parameters were: dry density, 2.31 g/cm³; and dry and saturated uniaxial compressive strengths, 31.56 MPa and 17.62 MPa, respectively. The steel needle engraving test sample is uniformly processed to 100 mm × 100 mm × 100 mm and divided into two groups: dry and saturated. The 0.4 mm and 1.0 mm steel needles were scored with a 10 mm stroke test in dry and saturated WCS, respectively. To reduce the randomness of a single test and improve the reliability of the results, a 25 mm stroke was added.See Table 2 for specific design scheme

**Table 2:** Test scheme for steel-needle scribing characterization.

Working condition of steel needle	WCS working condition	Number of steel needles	Important notes
Tip diameter 0.4 mm; Stroke 10 mm	Desiccation	1	Each steel needle is only used in this working condition. A single steel needle carries out a group of continuous engraving, which fits the engineering background of long-term sliding wear of TBM failure center cutter. Record the original shape of the tool tip before testing; In case of brittle collapse of the tool tip and serious deviation of the trajectory during the test, the whole group shall be canceled and retested; Progressive passivation is reserved as an evolution phenomenon, and data is not eliminated. Scribe 25 mm and repeat 4 times for each working condition.
	Saturated water	1	
Tip diameter 1.0 mm; Stroke 10 mm	Desiccation	1	
	Saturated water	1	
Tip diameter 0.4 mm; Stroke 25 mm	Desiccation	1	
	Saturated water	1	
Tip diameter 1.0 mm; Stroke 25 mm	Desiccation	1	
	Saturated water	1	

Remarks: two WCS test pieces are for standby; Two 0.4 mm and 1.0 mm steel needles are for standby. The multiple continuous characterizations of the same steel needle in this test are used to reveal the wear evolution law under cumulative sliding, and are not regarded as completely independent parallel samples of statistics; The horizontal comparison between different working conditions is achieved by independent steel needles.

### Mechanism analysis of sliding rock failure under the failure of central cutter rotation

The TBM hob relies on the synergy between the cutter head and the cutter to complete rock breaking. Under the combined effects of extrusion, shear, and tension, internal microcracks gradually initiate, expand, and penetrate, ultimately breaking and peeling off.

**Fig 3:**
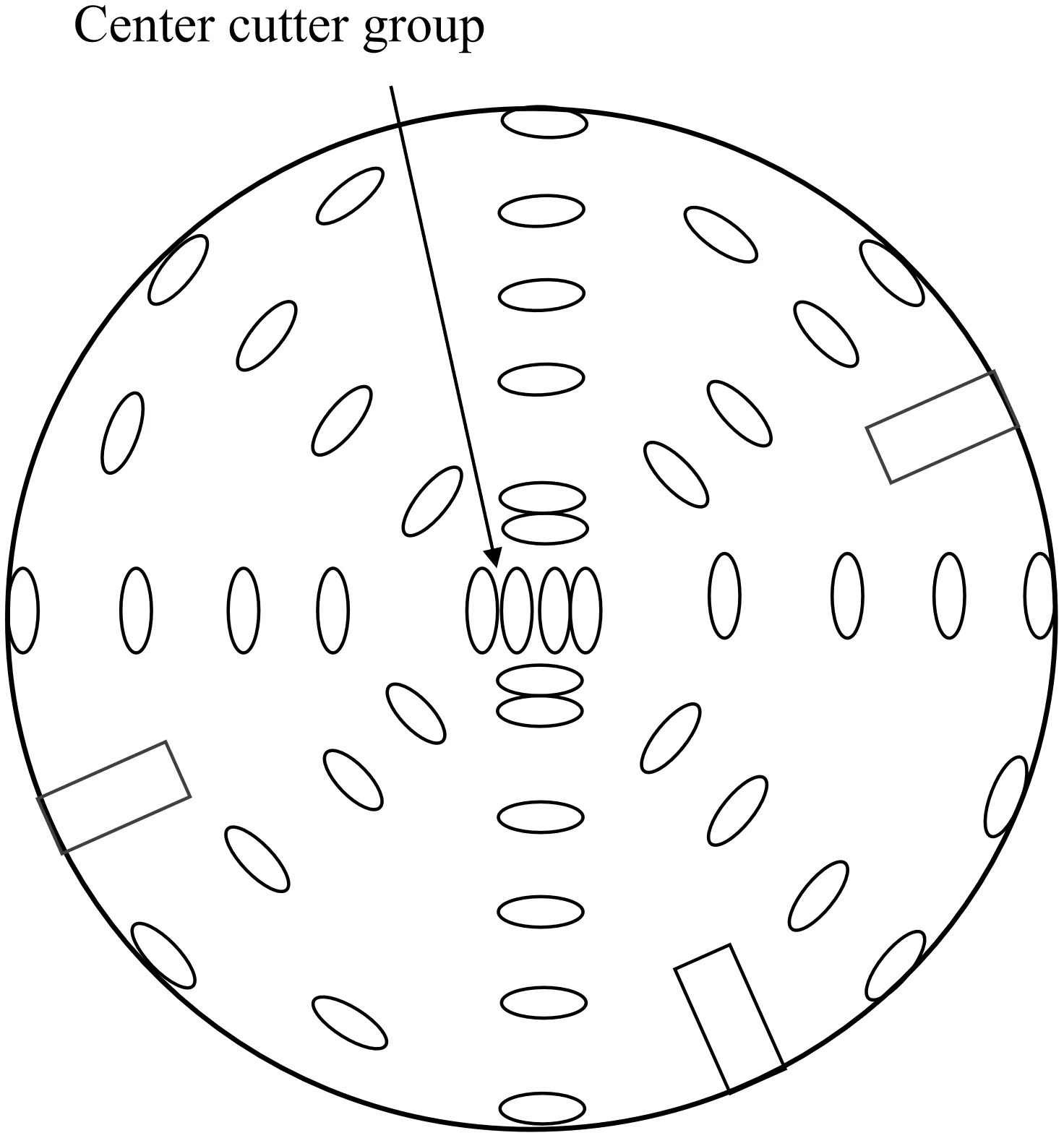
Schematic of cutter head with four cross-orthogonal double-disc center cutters.

According to the installation position, TBM hobs are divided into three categories: center hob, positive hob, and edge hob (Fig 3). Engineering practice has found that [31] when driving in water-rich strata containing clay minerals, rock-slag adhesion and mud-cake agglomeration are prone to occur in the central area of the cutter head, resulting in blocked rotation of the central hob, insufficient rotation, and even complete stagnation. The center cutter is close to the cutter head’s rotation center, and the linear speed of operation is low. The motion resistance due to the adhesion of the mud cake is superimposed, and the cutter’s rotation is clearly inhibited. Under normal driving conditions, the hob relies on continuous rotation to contact the rock mass. After the rotation is blocked, it is difficult for the tool to complete effective rotation, and a continuous relative slip forms between the tool and the rock surface, resulting in overall slip grinding.

Based on the above engineering characteristics, this article uses steel needle scratching tests to reproduce the sliding abrasion and rock breaking process after the failure of the central rolling cutter rotation. This experiment can reveal the core mechanical mechanism under this failure mode, providing a mechanistic reference for understanding the wear behavior of cutting tools on site, and is not directly equivalent to the full-scale TBM operating conditions. The specific analysis is as follows: (1) the steel needle used in the test is made of cemented carbide, which is consistent with the material of the TBM hob ring, and the hardness and wear resistance are matched with the engineering tools. Facilitating comparative analysis of wear mechanisms at the material level; (2) The results show that the steel needle and the rock sample surface form a continuous relative slip, and the controllable normal load can be applied at the same time, which can restore the essential characteristics of sliding friction and wear between the central hob and the rock mass in the state of rotation failure; (3) The steel needle and the rock sample are in point line contact, and the local stress concentration in the contact area is significant, which can accurately act on the rock particle gap and convex structure. During sliding, the shear effect at the contact surface is intense, and adhesive and abrasive wear are likely to occur. With the help of this experiment, quantitative observation results of the evolution of needle tip morphology under different working conditions can be obtained for inter group comparative analysis; (4) Using a steel needle tip as a simplified test component, referring to the 8 20 mm blade width range of the engineering disc rolling cutter, two types of needle tip blade widths of 0.4 mm and 1.0 mm were selected according to the geometric scaling ratio for testing. The observed evolution of needle tip morphology in steel needle engraving can provide a mechanistic reference for analyzing the wear characteristics of rolling cutters on site, and there is an undeniable scale effect between the two.

### Test results and analysis

#### XRD and SEM microscopic test.

**Fig 4:**
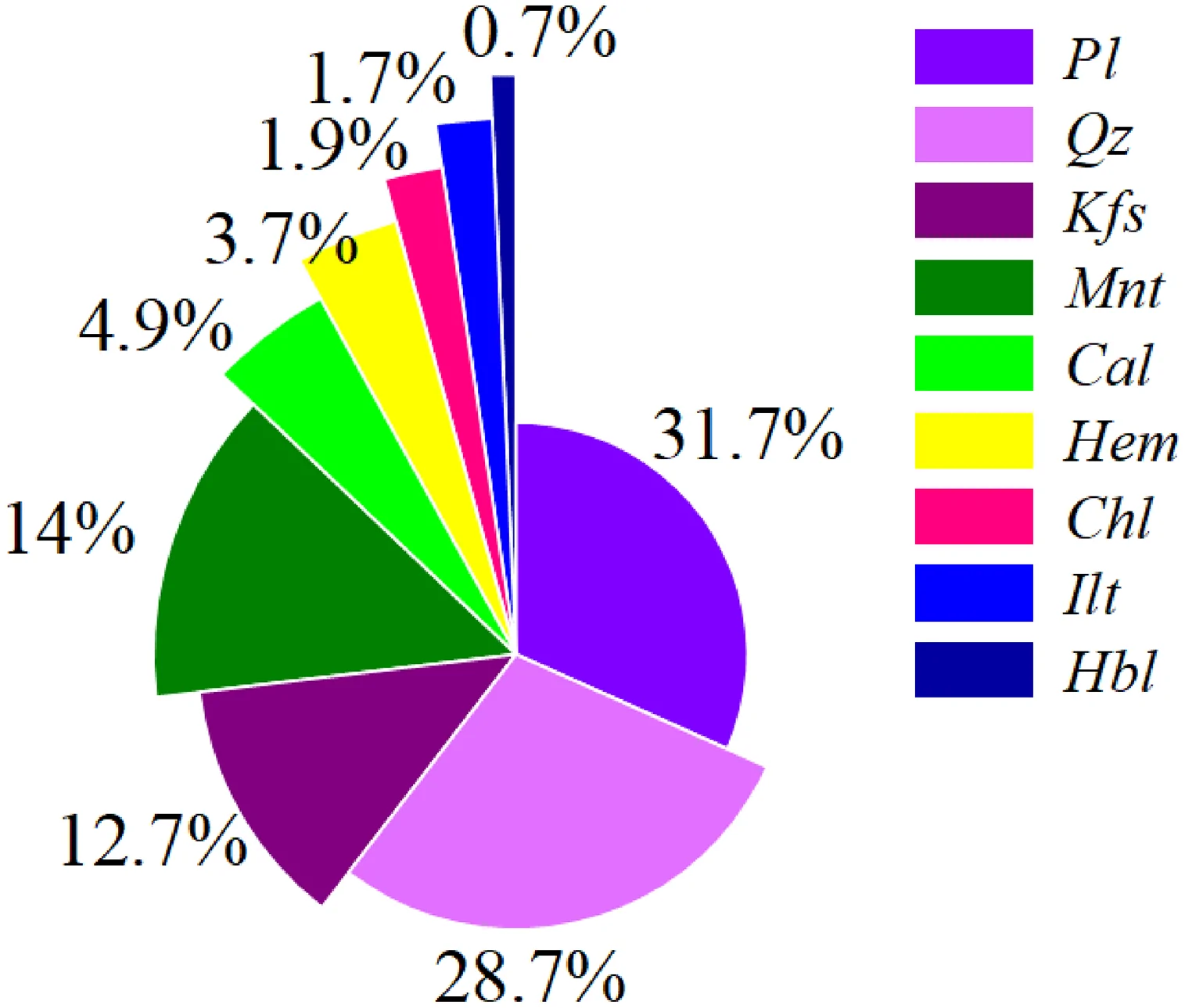
XRD test results of sandstone.

**Fig 5:**
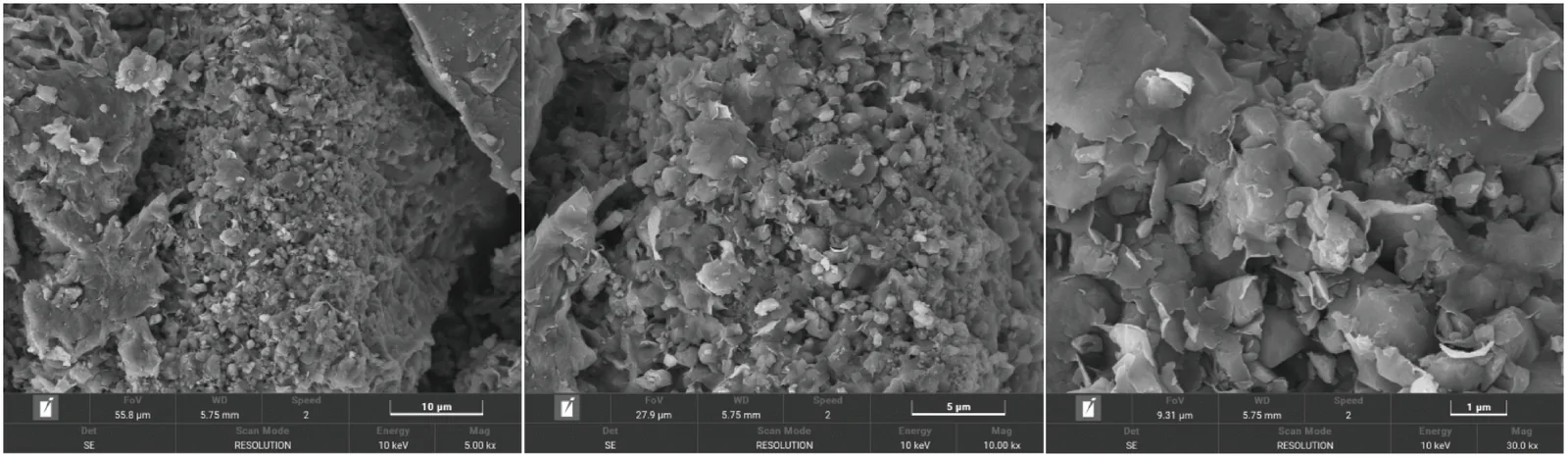
SEM image (magnified 5000, 10000, 30000 times).

XRD (X-ray diffraction) relies on the interaction between X-rays and the atoms in a material’s crystal structure to form a characteristic diffraction pattern, which can accurately characterize the mineral and phase composition of rocks. The XRD testing of sandstone samples was carried out using an XRD-6000 X-ray diffractometer for mineral composition detection, with a scanning range of 5° to 70°, a scanning step size of 0.02°, and a scanning rate of 2°/min. Based on Rietveld full spectrum fitting method, mineral quantitative phase analysis is carried out to obtain the mass proportion of various minerals in the sample, which is used to characterize the mineral composition characteristics of WCS. As shown in Fig 4, the main mineral component of WCS is quartz, containing skeletal minerals such as plagioclase and potassium feldspar; Simultaneously present are clay minerals such as montmorillonite, illite, and chlorite, as well as small amounts of calcite, hematite, and amphibole. Clay minerals are the main bonding components between sample particles, among which montmorillonite has strong hydrophilicity and is easily softened under water containing conditions, affecting the macroscopic fracture characteristics of rocks. SEM scanning electron microscopy, with its advantage of nanoscale high-resolution imaging, can intuitively observe the microscopic features such as particle arrangement, pore development, and bonding structure on the surface of sandstone [32,33]. This WCS used SU8020 scanning electron microscope (SEM) to observe the microstructure of sandstone. Before SEM observation, the rock sample powder was sprayed with gold for conductive treatment, and three-level magnification was set for observation. The 5000 fold image was used to identify the distribution characteristics of sandstone skeleton particles, intergranular pores, and cement; The 10000 fold image is used to capture the edge of plow furrows, microcrack propagation, and transitional features of debris adhesion, filling the visual gap between high and low magnifications; 30000 fold image focusing on the microscopic morphology characteristics of clay minerals, observing and selecting representative fields of view for multiple sets of samples. The observation results are shown in Fig 5.

From the SEM micrograph in Fig 5, it can be seen that the sandstone used in this test has developed an internal pore structure, and clay minerals are the main argillaceous cementation medium between particles, forming a typical weakly cemented porous rock mass as a whole. The microstructure characteristics are the fundamental internal factors that determine the mechanical response and surface anti-wear properties of sandstone. In the dry state, the clay cementation structure is intact, the particles are closely bound, and the rock mass is highly stable. After water saturation, water fills the voids, weakens cementation between particles, and reduces the rock mass’s stiffness and deformation resistance. At the same time, surface rock cuttings are prone to becoming muddy when exposed to water and are prone to forming argillaceous substances at the contact surface.

### The phenomenon of “mud cake” in the characterization of saturated sandstone

**Fig 6:**
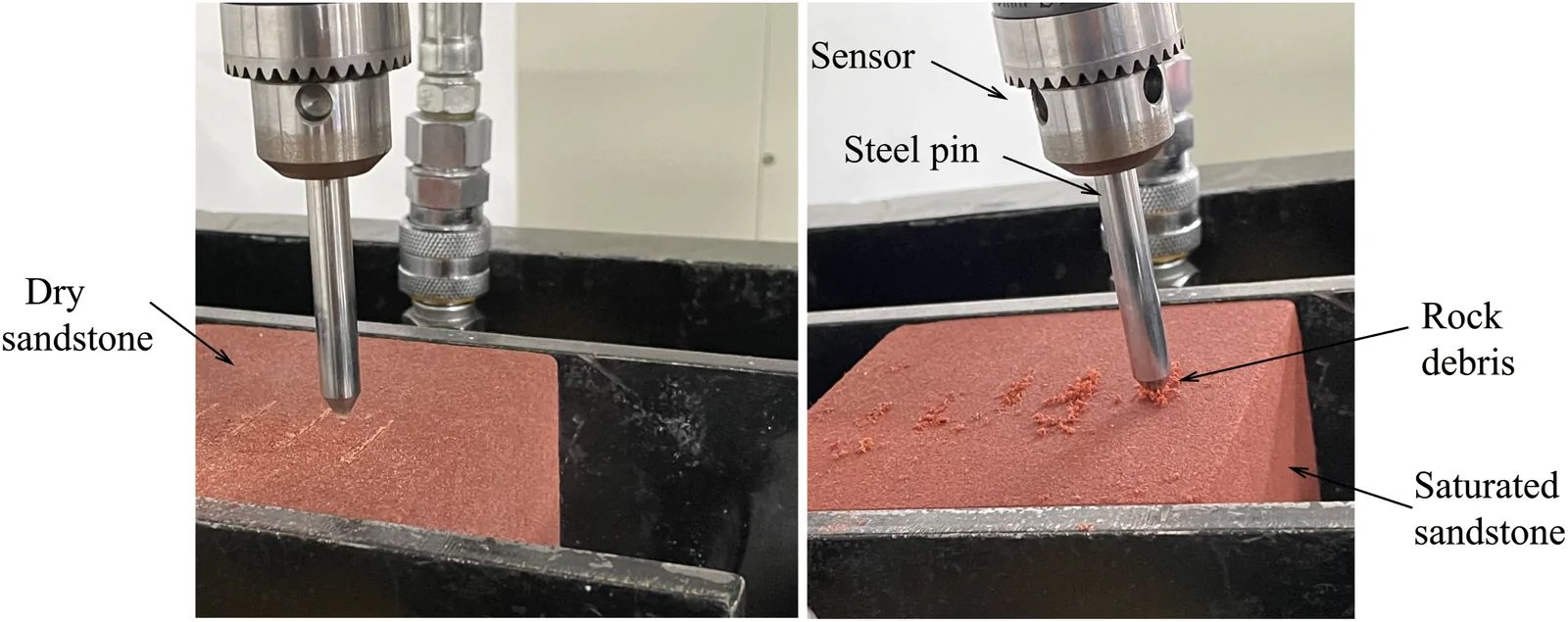
Steel needle wrapped by mud cake and rock slag of hob.

The clay and argillaceous cement in WCS are easily softened and dispersed when exposed to water. The rock powder produced by rock breaking is mixed with water to form a viscous mud cake, which firmly adheres to the hob ring and the surface of the hob body. In the test of cutting saturated sandstone with a steel needle, the inclusion slag appeared outside the tip of the needle (see Fig 6), which revealed the microscopic characteristics of rock slag that easily adhered and gathered when the saturated WCS was broken, and just provided evidence for the microscopic origin of mud cake formed by the TBM hob in this layer.

XRD tests on the WCS specimen in this study show that the total clay-mineral content is 17.6%, including 14% montmorillonite, 1.7% illite and 1.9% chlorite. Under water-saturated conditions, montmorillonite, a strongly swelling mineral, undergoes water-induced swelling, interface softening and particle peeling. It agglomerates with quartz and feldspar debris and gradually forms mud cakes [34]. The mud cake takes swelling clay minerals as the cementing phase and rock-debris particles as the filling phase. Water-saturated conditions enhance the adhesion capacity of mud cakes, making them more prone to wrap the cutter and further intensify the secondary abrasive-wear effect. Hard rock debris entrapped inside mud cakes on TBM centre-cutters can induce abrasive wear. Repeated scraping of the metal cutter-ring surface by rock debris gives rise to tool wear. Fine and cohesive slag particles generated from completely weathered argillaceous sandstone tend to mix with mud cakes, blocking the gaps between cutter-rings and the fitting interfaces between cutter-seat and cutter-ring, and thereby triggering sliding friction [35]. The above-mentioned wear mechanism distinguishes weak-cemented sandstone from hard rock formations dominated by primary abrasive wear only. Fig 6 shows rock-debris fragments adhered to the steel-needle surface, with obvious tip-adhesion behaviour. This phenomenon may be jointly controlled by clay-mineral hydration swelling and mechanical shear weakening of rock matrix. Limited by the present test conditions, the individual contribution of each factor cannot be distinguished. This paper only analyses tip-morphology-evolution behaviour under their coupled effect, and quantitative separation of their respective influences needs further experimental investigation in future work.

### Characterization of frictional resistance thermodynamic diagram

**Fig 7:**
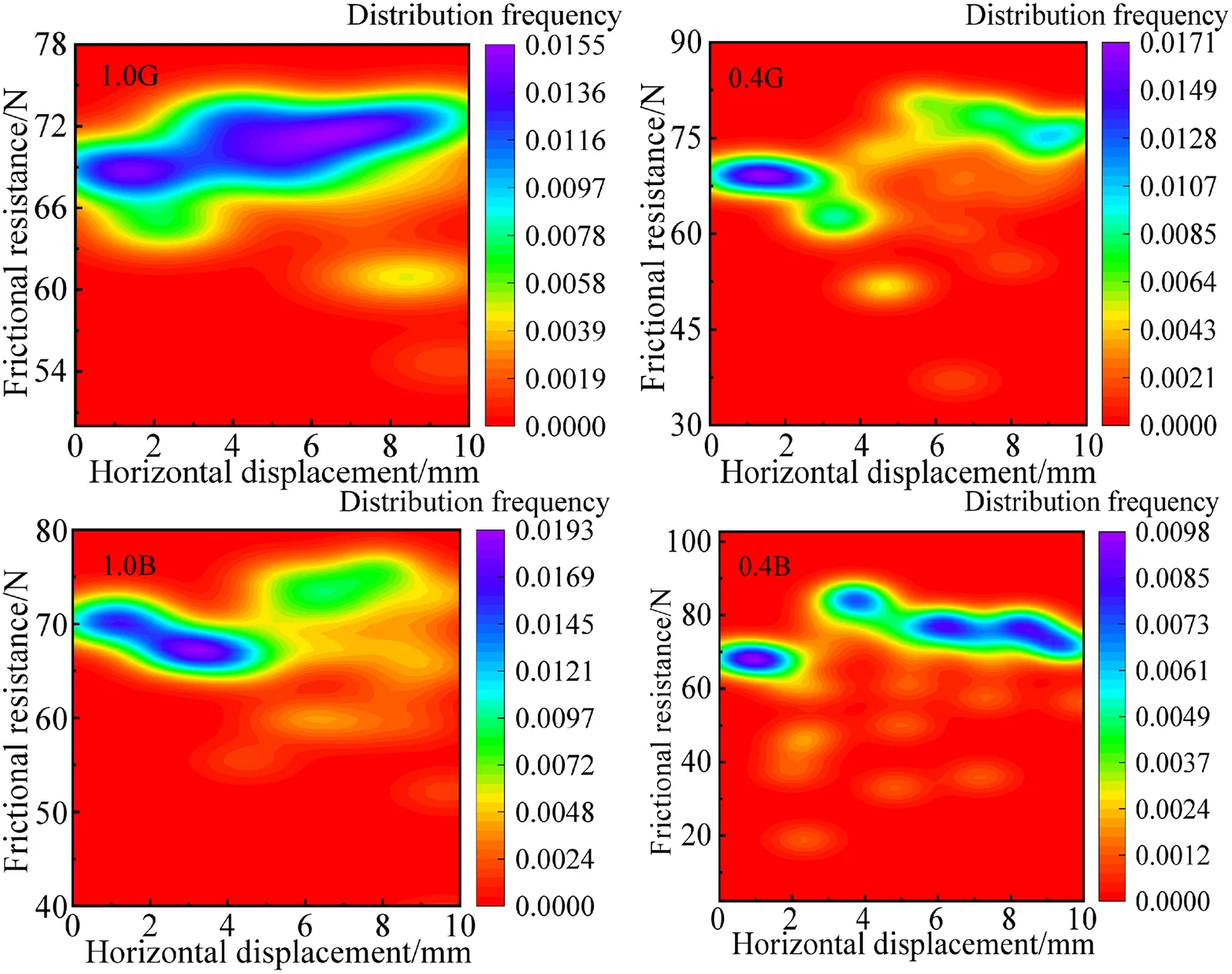
Thermal diagram of sandstone friction.

The thermal map shows the data distribution via a color gradient, effectively revealing the distribution law of friction. Fig 7 is the thermal diagram of friction resistance for different steel needle specifications, depicting sandstone with a stroke of 10 mm.

It can be seen from Fig 7 that the friction resistance of the 1.0 mm steel needle is stable at 65 N ~ 75 N under dry and saturated conditions; the fluctuation range of the friction resistance for the 0.4 mm steel needle is 60 N ~ 85 N, and the fluctuation characteristics are very significant.

Based on the equivalent mechanism analysis of the control test and the engineering prototype, in the weakly cemented sandstone formation, the friction resistance fluctuates greatly when driving with a small-edge-wide hob, which is not conducive to the stability of the driving system. The alternating friction load repeatedly causes the tool’s contact surface to experience impacts, thereby aggravating wear.

### Research on the deep evolution phenomenon

**Fig 8:**
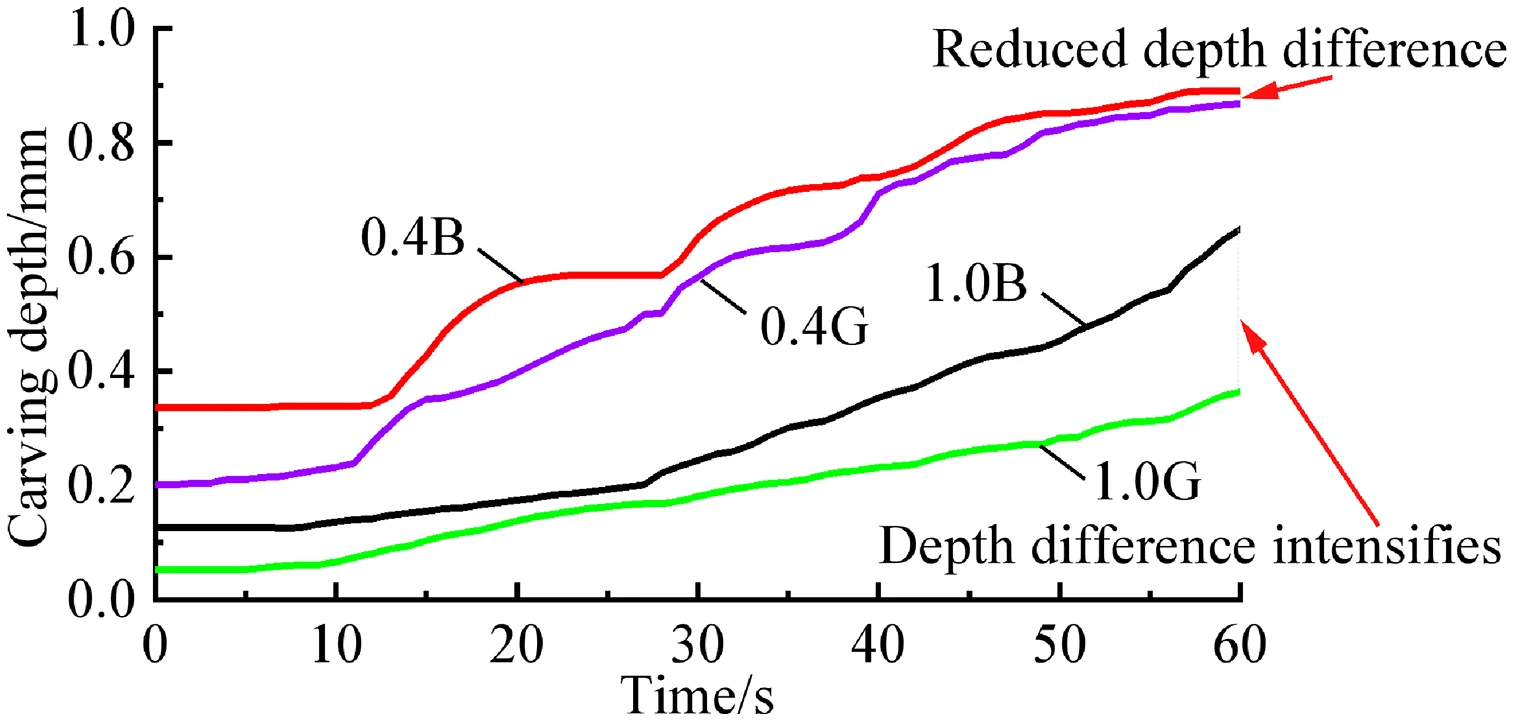
Depth time relationship of sandstone delineated by steel needle.

Fig 8 shows the variation curve of scoring depth of steel needles with different diameters in dry and saturated sandstone during the scoring process with a horizontal displacement of 10 mm and a time length of 0 ~ 60 s.

Fig 8 shows that the scratching depth of both 0.4 mm and 1.0 mm steel needles increases gradually with the scratching process for both dry and water-saturated sandstone. The observed behavior suggests that pre-existing damage fractures induced by scratching may pre-weaken the ahead WCS rock mass, possibly reducing subsequent penetration resistance and facilitating the continuous increase of scratching depth.

Under both dry and water-saturated conditions, the scratching depth of the 0.4 mm steel needle is larger than that of the 1.0 mm steel needle. Under identical constant normal load, the 0.4 mm needle has a smaller contact area and higher contact compressive stress, which facilitates damage to the cemented structure of WCS and yields a larger scratching depth. The scratching depth of water-saturated WCS is greater than that of dry specimens. The observed behavior suggests that water saturation may weaken the clay-based cemented structure within WCS, which may reduce rock penetration resistance and produce a larger scratching depth under the same load.

Under a constant normal load of 70 N, the 1.0 mm steel needle produces relatively low contact stress and shallow scratching grooves. The restraining effect from rock lateral confinement is limited, and the cementation strength of WCS dominates the scratching performance. As the steel needle continuously cuts WCS, the difference in scratching-depth increment induced by rock-strength discrepancy between dry and water-saturated conditions accumulates with scratching distance. Previous studies [36,37] have reported that under identical load, cutters with larger blade width produce higher total rock-chip yield and tend to accumulate clay mud films. The observed behavior suggests that a continuous clay adhesion layer may form on the needle surface under water-saturated conditions, which may reduce interfacial frictional resistance and promote larger scratching depth. In dry conditions, such clay adhesion layer is absent, leading to higher frictional resistance and restrained growth of scratching depth. Combined effects of the above two factors result in a continuous increase of scratching-depth difference between dry and water-saturated specimens for the 1.0 mm needle with scratching progress.

### Verification of steel needle engraving parallel test

#### Characterization of wear resistance displacement characteristics.

**Fig 9:**
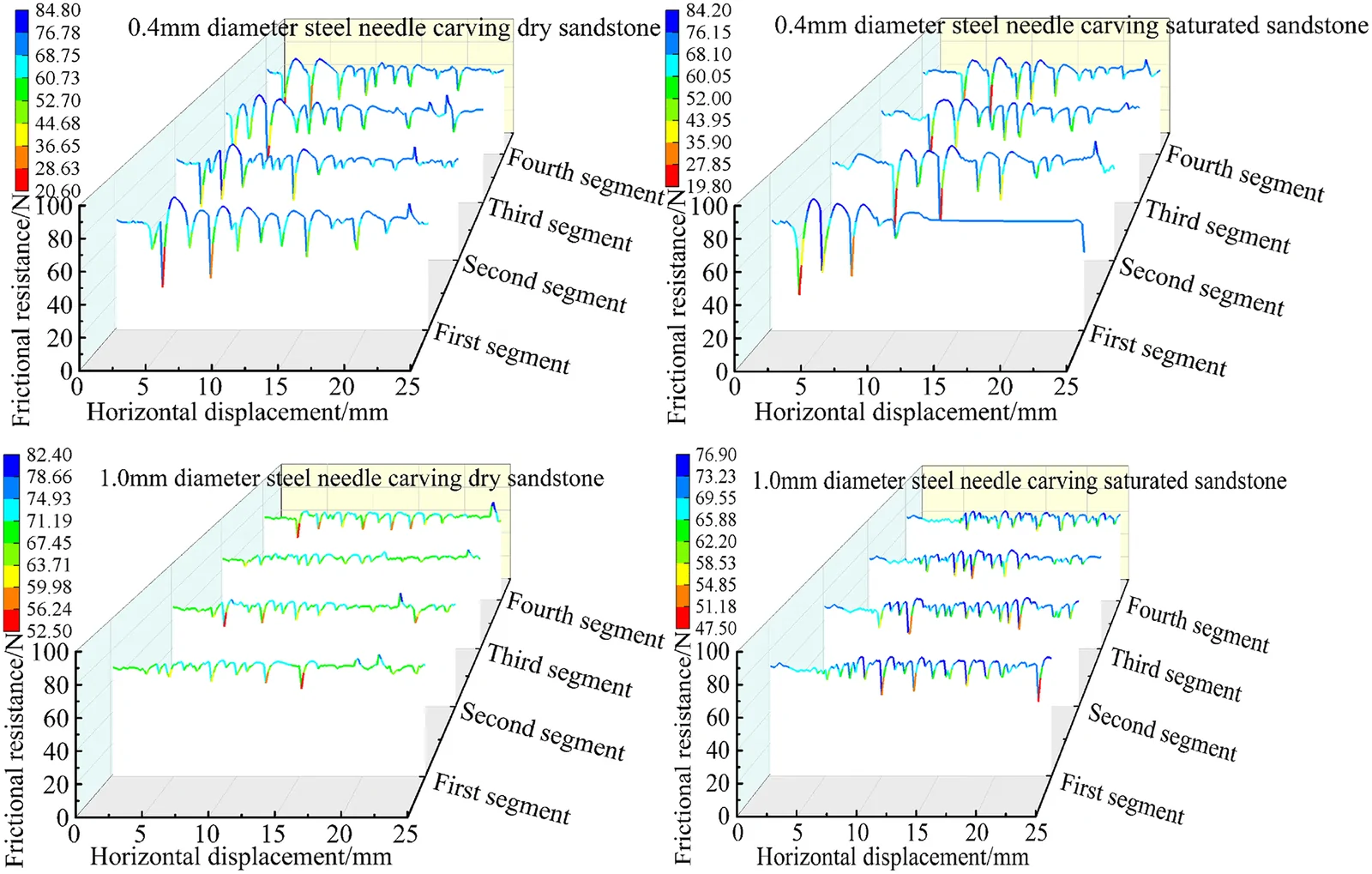
Time series curve of displacement and frictional resistance of sandstone carved by a single steel needle in four consecutive sections under various working conditions.

Fig 9 shows that the 0.4 mm and 1.0 mm steel needles are used to carve dry and saturated sandstone samples, forming three-dimensional changes of friction resistance corresponding to four 25 mm scribed strokes, which can directly reflect the changes of friction resistance of different specifications of steel needles and different water-bearing rock samples in the extended scribed stage.

Fig 9 shows the time series curve of the displacement frictional resistance of the same steel needle in four consecutive segments, with each segment corresponding to a cumulative slip distance of 25–100 mm, used to reveal the evolution characteristics of frictional resistance during the cumulative wear process. This result is a single sample time series repeated measurement and does not involve discrete statistical analysis of multiple parallel samples.

From the analysis of Fig 9, it can be seen that: (1) Comparing the curves of two different diameter steel needles, regardless of saturated or dry sandstone conditions, the fluctuation of the friction displacement curve of the 0.4 mm steel needle (simulating a small blade width rolling cutter) is significantly higher than that of the 1.0 mm steel needle in the initial stage of each section of the engraving. This phenomenon reflects that narrow diameter needles are more prone to stress concentration and are more sensitive to heterogeneous responses in sandstone media, resulting in alternating loads that exacerbate local impact effects. The characteristics of this indoor test are consistent with the conclusion of engineering literature [18], that is, the wear resistance of small blade wide rolling cutters is relatively weak, which is not conducive to long-term stable rock breaking; It should be noted that steel needle engraving is a simplified experiment and differs from the actual rock breaking conditions of TBM cutters. It only provides reference at the mechanism level. At the same time, comparing the dry and saturated subgraphs, it can be seen that under saturated conditions, the oscillation amplitude of the curve is further amplified, reflecting the wave effect of the narrow blade cutting tool’s stress amplified by the water content in the rock mass.

(2) For a 0.4 mm steel needle, the fluctuation amplitude of frictional resistance significantly decreases after each segment of engraving displacement exceeds 10 mm, and the curve gradually converges to a fluctuation level similar to that of a 1.0 mm steel needle. This feature suggests that the contact state at the tip of the needle evolves with the scratching process: in the initial stage of the narrow needle, the stress is highly concentrated, and as the scratching progresses, the tip of the needle may become dull, the equivalent contact range increases, and the stress at the contact position tends to be uniform. In contrast, the initial contact area of the 1.0 mm steel needle is larger, and the stress concentration effect is already weaker, so the overall frictional resistance during the entire engraving process is relatively stable. This result indicates that the blade width significantly affects the force characteristics by changing the contact area and contact stress state, and is an important parameter affecting the evolution of tool friction and wear.

To further quantify the average level of frictional resistance in each scoring section, Table 3 was obtained through statistical analysis.

**Table 3:** Friction parameters for segmented continuous scribing of single steel-needle.

Operating condition characteristics		0.4G	0.4B	1.0G	1.0B
average value (N)	1st segment	69.996	69.726	69.990	69.930
	2nd segment	69.761	69.696	69.901	69.737
	3rd segment	69.794	70.118	70.041	69.984
	4th segment	69.936	70.105	70.152	69.717
Overall average value(N)		69.872	69.911	70.021	69.842

Note: The four-segment scribing data correspond to sequential time-series measurements obtained from one single steel-needle specimen per working condition, and these segments shall not be treated as statistically independent parallel replicates. The overall average value is calculated from sequential segmented data of the identical needle, and it does not represent the mean result of multiple independent replicate specimens.

According to Table 3, during the continuous cutting process of a single steel needle in four sections, the average frictional resistance of each group ranged from 69.696 to 70.152, with small overall fluctuations and no significant abrupt changes. As the number of engraved segments increases, the evolution of frictional resistance shows a differentiated characteristic: the frictional resistance increases in the later stages of the 0.4B and 1.0G operating conditions, while it decreases and falls in the later stages of the 0.4G and 1.0B operating conditions.The steel needle engraving test shows that as the engraving distance increases, the frictional resistance can exhibit different evolutionary trends of rising or falling.

**Fig 10:**
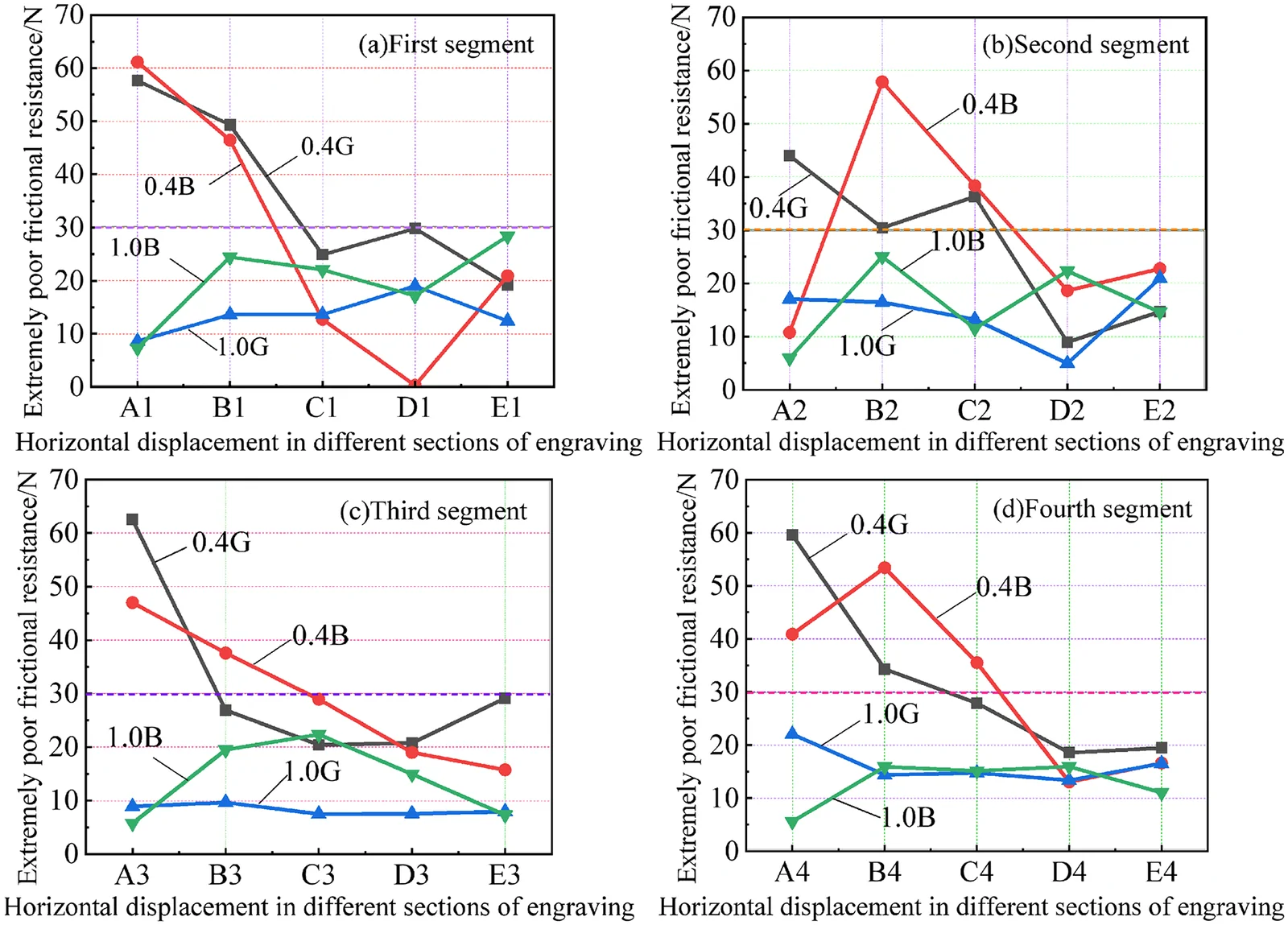
Range of horizontal displacement-frictional force during steel needle scratching under different times.

To visually present the characteristics of friction fluctuations under different working conditions and sections, Fig 10 is shown alongside the test values. Fig 10 shows the results of four marking cycles on sandstone with steel needles under the working conditions of 0.4G, 0.4B, 1.0G, and 1.0B. The length of each marking is 25 mm, which is divided into five sections (A ~ E), each of which is 5 mm. Each marking adopts a new path to avoid repetition. The ordinate is the range of frictional resistance reflecting the fluctuation, and the abscissa is the section (A ~ E) under different working conditions.

It can be seen from Fig 10 that the maximum range of 0.4G (A1, A2, A3, A4) and 0.4B (A1, B2, A3, B4) groups is 42–65 N, while the maximum range of 1.0B and 1.0G in sections A and B is ≤ 30N. This is consistent with the operating conditions of a TBM small-edge-width center cutter, which is prone to stress concentrations and severe stress fluctuations (large-edge-width cutters are more stable). Table 1: Engineering verifies that the small-edge-width center cutter is more prone to eccentric wear, edge collapse, and other failures.

Mark the D and E sections in the later stage, with all ranges below 30 N. the continuous cutting disturbance of the tool in the early stage is prone to form a dense damage layer on the surface of the rock mass, making the later rock breaking mechanical response more stable; This rule can provide experimental reference for TBM excavation in the rich water WCS formation, using the central cutter to pre construct a free face to improve the subsequent rock breaking conditions of the rolling cutter, optimizing the cutter arrangement to weaken the later load fluctuations.

### Statistical analysis of the scoring wear of the steel needle

The contact between the steel needle tip and the sandstone is described by the Hertz sphere plane contact theory [35]. Under the same 70N normal load, the smaller the radius of curvature of the needle tip, the higher the contact stress, and the stress of the 0.4 mm needle tip is significantly higher than that of the 1.0 mm needle tip. When the stress exceeds the yield strength of the steel needle, plastic deformation at the tip increases the diameter; under low stress, the loss of abrasive cutting material is the main reason.For each working condition, a single steel needle is used to carry out four consecutive scoring sections. After each scoring section is completed, the diameter of the needle tip is measured, and the measurement results are shown in Table 4.

**Table 4:** The measured diameter of the needle tip of a single steel needle after each continuous scoring segment under different working conditions.

Working condition		0.4G	0.4B	1.0G	1.0B
Initial diameter (mm)		0.401	0.397	0.993	1.040
Measured steel needle Diameter (mm)	Segment‑1	0.410	0.409	0.968	0.983
	Segment-2	0.416	0.415	0.961	0.977
	Segment-3	0.424	0.426	0.955	0972
	Segment-4	0.430	0.439	0.947	0.968

The above experimental observation results indicate that the initial diameter of the steel needle tip is a key factor affecting the evolution characteristics of the needle tip. After segmented scraping, the apparent diameter of the 0.4 mm steel needle tip shows an overall increasing trend, which can be attributed to the combined effect of plastic deformation of the needle tip and adhesion of rock debris and abrasive particles. Based on this, it is speculated that the loss of the steel body is relatively weak under this working condition; After scratching with a 1.0 mm steel needle, the apparent diameter of the needle tip shows a decreasing trend, indicating that the needle tip is mainly worn away by steel abrasion. The two types of needle tips exhibit completely different evolutionary characteristics. The observation results of the apparent diameter of the needle tip under various operating conditions are shown in Table 5.

**Table 5:** Changes in the apparent diameter of a single steel needle tip after each continuous scoring segment under different operating conditions.

Working condition		0.4G	0.4B	1.0G	1.0B
Initial diameter (mm)		0.401	0.397	0.993	1.040
Incremental diameter change (mm)	Segment‑1	0.009	0.012	0.025	0.057
	Segment-2	0.015	0.018	0.032	0.063
	Segment-3	0.023	0.029	0.038	0.068
	Segment-4	0.029	0.042	0.046	0.072
Final diameter change (mm)		0.029	0.042	0.046	0.072

**Fig 11:**
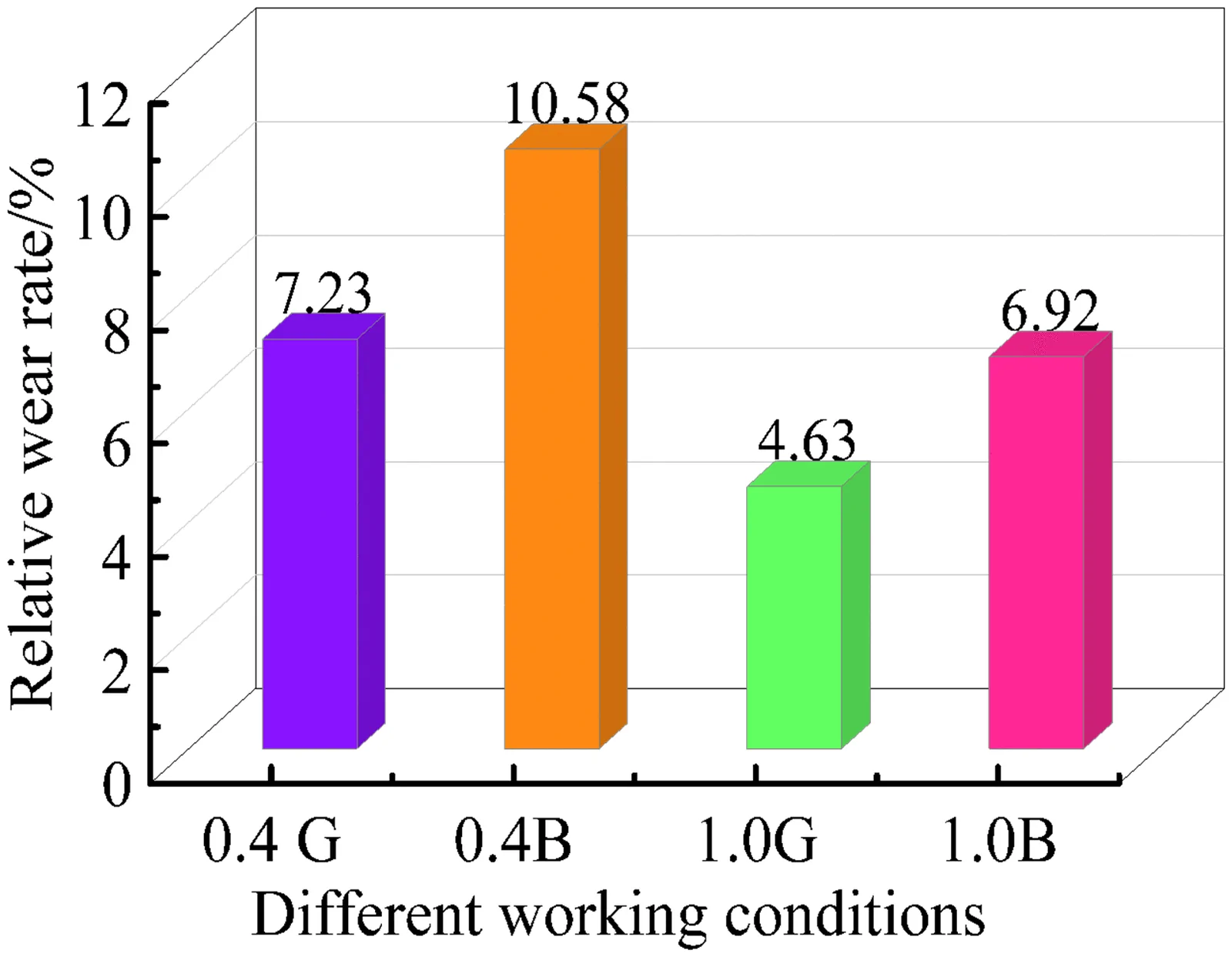
Relative wear rate of steel needle under various working conditions.

**Fig 12:**
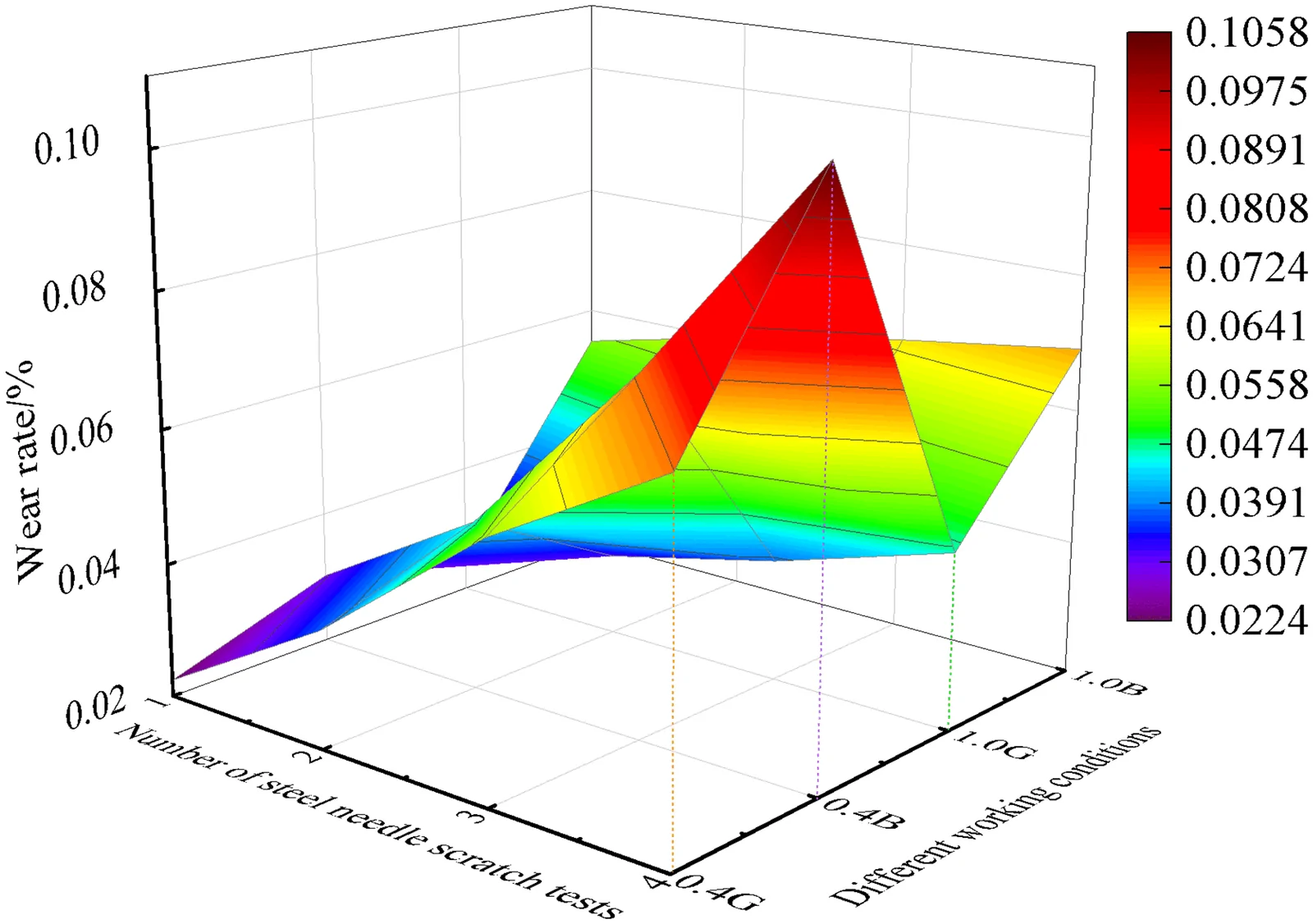
Wear rate of steel needle under various working conditions and test times.

From the observation results in Table 5, it can be seen that the total shape variation of the needle tip under 0.4B condition is 0.042 mm, which is 1.45 times that of 0.029 mm under 0.4G condition; The total variation of the needle tip under 1.0B operating condition is 0.072 mm, which is 1.57 times that of 0.046 mm under 1.0G operating condition. From the observation results of a single sample in this group, it can be seen that the change in needle tip morphology under saturated conditions is greater than that under dry conditions, indicating that the water containing environment of the surrounding rock will exacerbate the evolution of needle tip morphology. To visually display the wear observation results of each segmented steel needle after engraving, please refer to Fig 11 and 12. The sharp deep red high-value area in Fig 12 corresponds to the needle tip morphology state after completing the fourth segment of engraving under the 0.4B operating condition.

It can be observed from Fig 11 that the tip‑apparent‑diameter variation rate under condition 0.4B is 10.58%, approximately 1.53 times that of 6.92% under condition 1.0B. These observations reflect that the 0.4 mm‑tip condition is more sensitive to morphology evolution induced by water saturation. As shown in Fig 12, the growth amplitude of tip‑apparent‑diameter variation rate with increasing scribing segments is larger under saturated conditions than under dry conditions. Compared with larger‑size tips, small‑diameter steel‑needles exhibit more prominent morphological responses induced by saturated sandstone.

**Fig 13:**
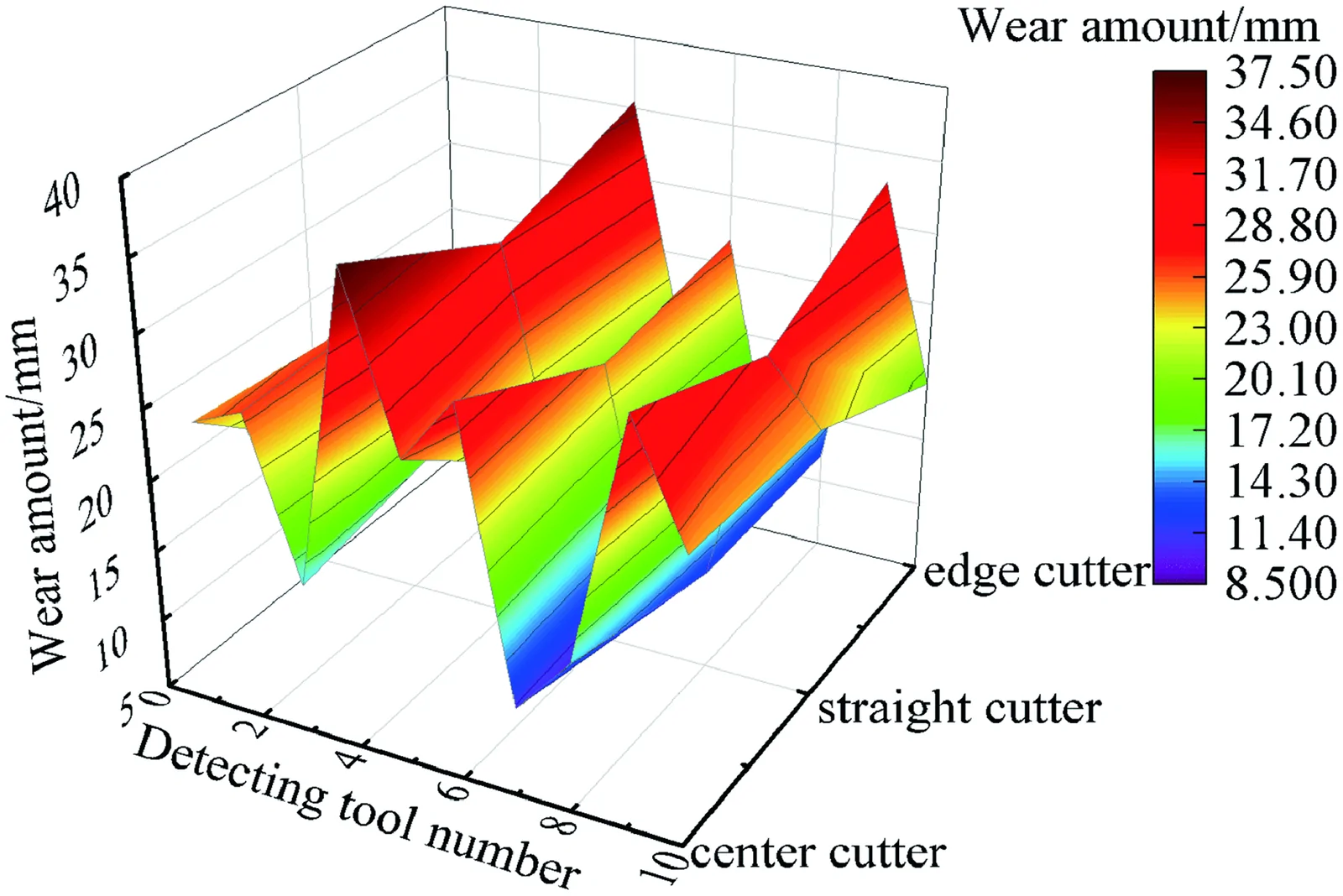
Measured wear data of partial disc cutters for shield machine on Shenzhen Metro Line 9.

Fig 13 presents the 3D cloud map plotted based on hob-wear monitoring data of shield cutters from Shenzhen Metro Line 9 reported in Reference [36]. The deep-red regions in the Fig denote the maximum wear magnitude, showing that the centre-cutter suffers more severe wear than ordinary scrapers. This engineering phenomenon can be cross-referenced with the TBM-engineering survey statistics in Table 1 of this paper. The centre-cutter is prone to suffer intensive sliding friction and stress concentration, which makes it more susceptible to severe wear.

### Derivation and verification of the sliding wear formula of the center cutter

To investigate the influences of water content and cutter‑blade width on sliding‑wear behaviour of rotation‑failed centre hobs in weak‑cemented strata, formula derivation for centre‑hob sliding wear is carried out based on classical wear theory and mechanical definitions of rock hardness. A mechanical model for the contact section between cutter and sandstone is established according to the mechanical relationship of material hardness, and the quantitative relationship of wear response is derived combined with the fundamental abrasive‑wear formula. On this basis, key correction parameters of water content and blade width are introduced, and an empirical correlation for blade‑edge morphology evolution of centre hobs in weak‑cemented sandstone strata is obtained. It can provide theoretical references for interpreting the sliding‑wear mechanism of centre hobs under rotation‑failure conditions.

To clearly define the physical meaning and dimension of each parameter in the derivation process, all symbols involved in this derivation are summarised in Table 6.

**Table 6:** Meanings and functions of symbols for sliding wear formula of center cutter.

Formula	Parameter	Meaning	Role in derivation
(1)	*H*	Hardness of worn material, MPa	It is a basic parameter for abrasive wear calculation to characterize the ability of materials to resist local plastic deformation
(1)	*F*_*N*_	Normal contact load, N	Determine the contact pressure
(1)	*A*	Plastic actual contact area, mm^2^	Effective area of plastic contact under normal load, hardness defines the core parameter
(2)	*W*_*V*_	Theoretical volume wear amount, mm^3^	Material volume loss caused by abrasive sliding
(2)	*K*	Wear coefficient (dimensionless)	Comprehensively reflect the characteristics of abrasive particles and wear efficiency, which is obtained by test calibration
(2)	*s*	Sliding length of abrasive particles relative to worn surface, mm	Relative sliding distance between cutting tool and rock mass
(4)	*k*_0_	Foundation wear coefficient (dimensionless)	Test acquisition to characterize the inherent wear mechanism
(4)	*k*_*s*_	Sliding abrasive wear coefficient (dimensionless)	Modified wear coefficient related to rock abrasiveness
(4)	*R*_*c*_	Uniaxial compressive strength of rock, MPa	Rock mechanics parameters to control the strength of rock mass abrasion ability
(4)	*Q*_*E*_	Equivalent quartz content of rock (dimensionless, decimal form)	Mineral composition index of rock abrasiveness
(5)	*N*	Cutter head speed, r/min	The rotating speed of TBM cutter head determines the tool sliding travel
(5)	*V*	Tunneling speed, mm/min	TBM forward speed
(5)	*D*_*0*_	Tool diameter, mm	Calculating the geometric parameters of the sliding path of the center cutter
(5)	*L*	TBM tunneling distance, mm	Geometric parameters of hob layout on cutter head
(5)	*f*	Slip correction factor (dimensionless)	Modify the sliding distance to characterize the low-speed sliding characteristics of the center cutter
(5)	*s*_*X*_	Sliding distance of center cutter, mm	Total relative sliding stroke between rock mass and central blade, core variable for wear calculation
(6)	*A*_1_	Contact area of cutter ring, mm^2^;	Effective contact area between tool and rock under load, wear volume derivation of basic parameters
(7)	*Δh*	Center cutte line wear, mm.	Radial wear depth generated by the centerline position of the central blade, visual wear observation index
(8)	*V*_*rock*_	Rock breaking volume of center cutter, mm3;	Establish correlation between excavation volume and wear depth
(10)	*ν*	Wear rate at the centerline of the cross section of hob blade, mm/m^3^	Linear wear depth of center cutter per unit rock breaking volume
(11)	*λ*	Wear amplitude ratio under saturated and dry conditions (dimensionless)	Correction factor considering water content effect of rock
(11)	*ω*	Moisture content,%	Mass water content of weakly cemented sandstone
(12)	*b*	Center cutter width, mm	Geometric dimension of tool contact edge feature
(12)	*R*_*i*_	Installation radius of center cutter, mm	Radial layout position of center cutter on cutter head

Note: Some parameters in this table are intermediate theoretical variables derived from classical wear theory, rather than quantities directly measured in the steel‑needle scribing tests.

See the above table for the complete definitions of all parameters involved in the subsequent derivation of this chapter. The following formulas will not list the parameter definitions one by one.

According to the definition of hardness, that is, the ability of the material to resist local plastic deformation, the following results are obtained:


$$\begin{array}{c} \text{H}=\frac{{\text{F}}_{\text{N}}}{\text{A}} \end{array}$$ (1)

The theoretical volumetric-wear relation from classical abrasive-wear theory is:


$$\begin{array}{c} \text{Wv}=\text{kAs} \end{array}$$ (2)

Substitute Equation (1) into Equation (2) to derive the theoretical relation for tip-volume change:


$$\begin{array}{c} \text{Wv}=\text{K}\frac{{\text{F}}_{\text{N}}\text{s}}{\text{H}} \end{array}$$ (3)

Based on the on-site data verification of Guangzhou Metro, Guangzhou Shenzhen Hong Kong Shiziyang Tunnel, and other projects in reference [27], it was found that the sliding abrasive wear coefficient is linearly correlated with the rock abrasiveness:Referring to the logarithmic fitting form of rock abrasivity proposed in the existing research [27], the basic wear coefficient K0 is introduced for correction, and the calculation formula of sliding abrasive wear coefficient is obtained


$$\begin{array}{c} k_{s}={\text{k}}_{\text{0}}\left[\text{0.8164ln}\left(\text{RcQE}\right)+0.1761\right] \end{array}$$ (4)

This formula refers to the existing TBM engineering rock mass attrition experience fitting form. *R*_C_ *Q*_E_ only uses the test measured value to participate in logarithmic calculation, and does not carry physical units. It is a conventional processing method for engineering experience fitting.

Introduce a sliding correction coefficient f to fit the low linear velocity sliding characteristics of the center blade. Based on the research of Han Bing Yu et al. [38], the sliding distance s of the central blade is simplified as:


$$\begin{array}{c} {\text{s}}_{\text{X}}=\frac{\pi {\text{D}}_{\text{0}}\text{NL}}{\text{V}}\text{f} \end{array}$$ (5)

Where: *πD*_0_ is the sliding perimeter corresponding to one revolution of the central cutter (mm/r); *NL/V* is the total number of turns of the cutter head in the whole process of tunneling, and the two are multiplied to obtain the foundation sliding distance. The sliding correction factor F is introduced to represent the reduction of the sliding distance caused by poor rotation.

Contact area of cutter ring:


$$\begin{array}{c} {\text{A}}_{\text{1}}=\text{b}\frac{\pi \text{D}0}{\text{2}} \end{array}$$ (6)

Line wear of center cutter:


$$\begin{array}{c} \Delta \text{h}=\frac{W_{\text{v}}}{{\text{A}}_{\text{1}}}=\frac{{\text{k}}_{\text{s}}{\text{F}}_{\text{N}}\text{S}}{\text{Hb}\pi \text{D}0\text{/2}} \end{array}$$ (7)

The rock breaking area of the central cutter is approximately cylindrical (the radius of the cylinder is the installation radius *R*i of the central cutter), and its rock breaking volume is:


$$\begin{array}{c} \text{Vrock}=\pi {\text{R}}_{\text{i}}^{\text{2}}\text{L} \end{array}$$ (8)

Wear rate of central cutting line v:


$$\begin{array}{c} \text{v}=\frac{\Delta \text{h}}{\text{Vrock}} \end{array}$$ (9)

$$\begin{array}{c} \text{v}=\frac{2{\text{K}}_{\text{0}}\text{f}F_{N}[0.8164ln(Q_{E}{\text{R}}_{c})+0.1761]}{\text{Hb}\pi {\text{R}}_{\text{i}}^{\text{2}}V}N \end{array}$$ (10)

Equation (10) indicates that a larger normal contact pressure *F*_N_ produces higher contact stress at the tool-rock interface. Harder rock corresponds to larger *R*_C_ and stronger abrasiveness; a larger *Q*_E_ means more energy is consumed for rock breaking, which aggravates abrasive cutting and apparent geometric alteration on the tool surface. Higher cutter-head rotational speed *N* leads to more frequent friction and impact between hob and rock, intensifying the degree of surface geometric variation per unit excavated rock volume. A smaller hob installation radius amplifies the sliding effect for hobs located in the central zone and aggravates their surface geometric alteration. The installation radius *R*_i_ of the central hob is relatively small; shear-dominated rock breaking and sliding contact become its dominant rock-breaking modes, eventually resulting in prominent apparent-diameter variation of the hob tip.

In the combined steel-needle test, the relative tip-diameter variation of the 0.4 mm steel needle (equivalent blade width b_1_ = 8 mm) scratching water-saturated WCS reached 10.58%. The corresponding relative tip-diameter variation of the 1.0 mm steel needle (equivalent blade width b_2_ = 20 mm) was 6.92%. A logarithmic fitting was performed for the relation *v* ∝ *b*^*n*^, yielding n ≈− 0.46, i.e., *v* ∝ *b* ^− 0.46^.

To quantify the aggravating effect of clay‑mineral swelling under water‑saturated conditions on tip geometric evolution, the relationship between tip‑geometric response and water content was established via steel‑needle scratching tests. According to test measurements, the water contents of dry and water‑saturated sandstone specimens are 0.5% and 6.62%, respectively. The relative tip‑diameter variation under saturated condition is 1.57 times that under dry condition, and linear fitting yields:


$$\begin{array}{c} \lambda =1+0.093\text{(}\omega -0.5\text{)} \end{array}$$ (11)

Field measurement data from a TBM project in Xinjiang indicate [16] that the installation radius of the central hob is considerably larger than that of the normal hob. The average tip‑diameter variation per unit excavated rock volume for the central hob reaches 0.1416 mm/m³, which is distinctly higher than the corresponding value of 0.0776 mm/m³ for normal hobs.This observed trend is consistent with Equation (11).

Considering the combined effects of water content and equivalent blade width, the sliding-action coefficient is modified. Based on the fitting results from steel-needle scratching tests, an empirical correlation for relative tip-diameter variation describing the interaction between central hob and weakly cemented sandstone is obtained:


$$\begin{array}{c} \text{v}=\frac{2{\text{K}}_{\text{0}}\text{f}F_{N}[0.8164ln(Q_{E}\sigma_{c})+0.1761]}{\text{H}\pi {\text{R}}_{\text{i}}^{\text{2}}V}N\left[1+0.093\text{(}\omega -0.5\text{)}\right]{\text{b}}^{\text{-0.46}} \end{array}$$ (12)

The steel‑needle scratching test results show that the relative tip‑diameter variation of the 0.4 mm steel needle is distinctly larger than that of the 1.0 mm steel needle. In Equation (12), the blade width is introduced in the form of negative‑power term b^− 0.46^, exerting the most prominent influence on the relative tip‑diameter variation. Under water‑saturated conditions, the relative tip‑diameter variation of the steel needle reaches 1.57 times that under dry conditions, and this effect is characterized by the independent correction term “1+0.093 (ω−0.5)” in the equation.

Equation (12) also reflects that the relative tip‑diameter variation is inversely proportional to the square of the hob installation radius. For hobs with smaller installation radius, once rotation failure occurs, the total relative sliding distance between the hob edge and surrounding rock becomes longer under identical tunnelling conditions. After the hob loses its normal rotation capacity, its self‑rotation cannot offset the tangential displacement induced by cutter‑head revolution. Consequently, the hob edge continuously scrapes the surrounding rock, and frictional dissipation energy concentrates at the tool‑rock contact zone. Higher frictional energy input per unit sliding length further aggravates the tip geometric alteration per unit volume of excavated rock. This experimental trend is generally consistent with field observations that central hobs under rotation‑failure status exhibit more prominent tip‑geometry change than normal hobs [17,18].

Combined with the formula structure and abrasive‑contact mechanism, blade width, water content and hob installation radius are the dominant sensitive parameters governing the relative tip‑diameter variation. For surrounding rock with high water content, strict control of tunnelling parameters (cutter‑head rotational speed *N*, advance rate *V*), enhanced muck conditioning [39] and mud‑cake prevention are recommended to suppress geometric deterioration induced by surface adhesion on the tool. For surrounding rock with low water content, adjusting tunnelling parameters and adopting a reasonable equivalent blade width can alleviate the geometric evolution of the tool tip.

The empirical relationship of tool wear derived in this paper is based on the experimental data of two kinds of needle tip diameters, 0.4 mm and 1.0 mm, and two kinds of water conditions, dry and saturated. The power term of blade width and humidity correction term are interpolation empirical factors within the range of test parameters, which are only applicable to the current test conditions, and belong to the preliminary empirical relationship; The applicability of the model under the conditions of intermediate tip diameter and multi gradient moisture content needs to be further verified by more subsequent working condition tests.

## Discussion

Compared with studies on rock breaking by conventional hobs, this paper emphasizes the abrasion characteristics of the center cutter under rotational failure and pure sliding, and the resulting law can serve as a reference for analyzing abnormal wear of the center cutter in similar strata.

### Key influencing factors and correlation between model and project

Blade width and water content are the core influencing variables of the tests, which directly dominate the apparent morphological evolution of central hob edges. Based on the steel-needle scratching test data, an empirical correlation is fitted with equivalent blade width and rock water content as the main variables. The variation characteristics of the correlation agree well with the test observations, which clarifies the physical mechanism and quantitatively reflects the parameter sensitivity of hob edge morphological changes.

### Consistency between the scoring test and the center cutter wear mechanism

**Fig 14:**
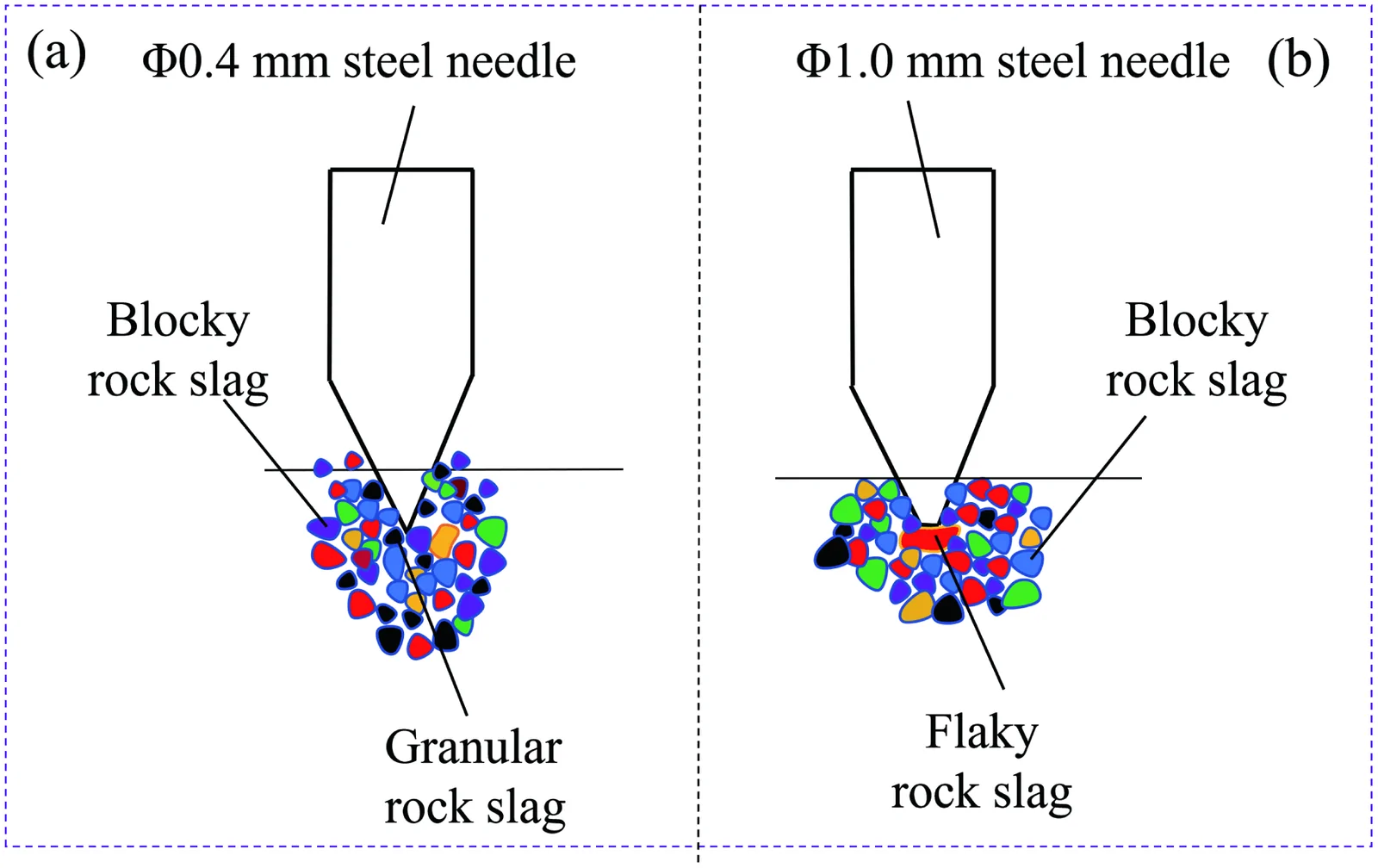
Characteristics of sandstone scratching by steel needles with different diameters.

In actual TBM construction, the rock-breaking conditions of the hob are highly complex, and it is difficult to replicate them in the laboratory. However, this test simulates the wear behavior of hobs to a certain extent. The conclusion on the factors affecting wear can serve as a reference for engineering practice: the rock slag morphology after the 0.4 mm steel needle simulating the center cutter with a small blade width and the 1.0 mm steel needle simulating the front cutter with a large blade width, as shown in Fig 14.

TBM tunnelling involves highly complex in-situ conditions that cannot be fully reproduced in laboratory tests. Nevertheless, the present tests can mimic the sliding contact behaviour between hob and rock under rotation-failure conditions, and the derived parameter-related findings can provide references for engineering practice. The rock-slag morphologies produced by steel needles of different equivalent blade widths are shown in Fig 14. Under the 0.4 mm small-blade-width needle (Fig 14a), rock debris is mainly granular, generated jointly by cutting, sticking-jumping and discontinuous friction. Severe stress concentration occurs beneath the needle tip; the local stress exceeds the compressive strength of water-saturated weakly cemented sandstone. The observed behavior suggests that this condition may promote abrupt rock-particle fragmentation, which leads to drastic fluctuation of frictional resistance. This observation is comparable to the large-amplitude resistance fluctuation of field central hobs suffering rotation failure. For the 1.0 mm large-blade-width needle (Fig 14b), flaky rock slag dominates, corresponding to a stable continuous sliding-friction mode with low frictional-resistance fluctuation. The contact area of the 1.0 mm steel needle is approximately 6.25 times that of the 0.4 mm one. Contact stress is significantly dispersed, only inducing near-surface plastic deformation of rock. Flaky slag slides along the contact interface, and the scratching depth is constrained by frictional boundary conditions.

The empirical correlation fitted in this study takes equivalent blade width and water content as core correction parameters, which is corroborated by the above-mentioned experimental observations. Blade width modifies contact area and interfacial stress, further governing friction modes and resulting in the granular-friction/ flaky-sliding slag patterns in Fig 14. Water saturation may promote damage to the cemented skeleton of sandstone and alter the abrasive response of rock mass. This quantitative correlation can mechanically interpret the evolution trends of relative tip-diameter variation, slag morphology and interfacial friction characteristics under various working conditions, and achieves effective combination of qualitative mechanism analysis and quantitative parameter characterisation based on laboratory tests.

### Scaling relationship and applicable limitations of the center cutter scaling test

The test uniformly applies a constant normal load of 70 N, and the load value is taken according to the published reference [11], which is also a general working condition recognized by the industry for the research on the scoring wear of steel needles, and is used for the parallel comparison of each working condition. In this paper, the 1:20 geometric correspondence is established, and the blade width of the 8 mm and 20 mm prototype center cutter is compared with the 0.4 mm and 1.0 mm steel needles respectively. Under the condition of uniform load, the change of contact width will form the differential dimensionless contact pressure (*P*/*R*_C_); This paper analyzes the rock contact mechanical response under the condition of contact stress change caused by blade width change, and explores the rock breaking and wear characteristics caused by the difference of contact state caused by blade width adjustment. This test scheme simulates the engineering characteristics of TBM central cutter rotation failure and relatively stable driving thrust under pure sliding friction conditions, focusing on the comparison of indoor multi condition mechanism, and does not rely on the scaling relationship to quantitatively predict the on-site tool wear. There are objective differences between the test and the field prototype in cutting speed, cuttings transportation, tool material and other aspects. The obtained laws are only used to reveal the evolution trend of tool rock interface wear.

This study only selected representative rock samples for XRD and SEM mineral-background characterisation, and did not conduct quantitative mineral detection on each scribing-test rock block one by one. Therefore, it is difficult to accurately evaluate the mineral-composition dispersion among different specimens. All test specimens were extracted from the same bulk rock block to control the fluctuation of mineral composition. This research focuses on the influences of blade width and moisture content on steel-needle tip-morphology evolution.

### Sensitivity of empirical correlation parameters and geological applicability

To investigate the impact of various input parameters on the output results of the indoor empirical correlation, a ± 10% perturbation analysis was conducted on key parameters. For the linearly correlated parameters *K*_*0*_*, f, F*_*N*_*, N*, increasing the parameters by 10% will synchronously increase the calculated rate of change in the apparent diameter of the needle tip by 10%; Reduce parameters by 10%, and synchronously reduce calculation results by 10%. For parameters H and V located in the denominator, increasing the parameter by 10% will result in a decrease of approximately 9.09% in the calculation result; Reducing the parameter by 10% results in an increase of approximately 11.11% in the calculation result. The blade width b has a power exponent effect of 0.446, with a 10% increase in blade width resulting in a 4.24% decrease in calculation results; The blade width decreases by 10%, and the calculated result increases by 4.94%. The moisture content correction term experiences an output change of approximately 0.47% due to a ± 10% perturbation in the parameters, indicating a relatively low sensitivity in this empirical relationship.

The above experience correlation is based on the fitting of indoor carving tests on weakly cemented sandstone in the Cretaceous system. The applicable working conditions are limited to quasi-static unidirectional sliding, self rotation failure center blade sliding and abrasion dominant scenarios, and the applicable blade width range is 0.4–1.0 mm. It is not suitable to directly apply it to hard rock, strongly cemented rock, and normal rolling cutting conditions of TBM cutters. When there are significant differences in the mineral composition and cementation characteristics of engineering rock masses, it is necessary to recalibrate the relevant input parameters.

### Research boundary and rationality

The steel-needle scribing test adopts constant normal load and unidirectional sliding load, which can characterise the sliding-cutting mechanical behaviour under the rotation-failure condition of TBM centre cutters. This work establishes an empirical correlation model for this specific failure condition, which does not cover the rolling-motion mode of centre cutters during normal operation and is only applicable to scenarios where sliding wear dominates. This scaled quasi-static test can reproduce sliding abrasion, contact-stress concentration, and rock hydration-softening effects at the cutter-rock interface, facilitating variable comparisons of blade width and moisture content; however, it cannot reproduce field engineering conditions such as rolling-sliding mixed contact, hob rotation, transient impact load, three-dimensional constraint of surrounding rock, and continuous discharge of rock debris, and cannot achieve full physical simulation of the prototype tool. The analysis of fragmentation and adhesion mechanisms in this study mainly relies on macroscopic experimental observations, and microscopic evolution characteristics need to be further revealed in subsequent work. In the future, we will expand experimental conditions and improve comparative investigations on tip-morphology evolution of TBM centre hobs under different motion states.

## Conclusion

The steel‑needle scratching test can reproduce partial tool‑rock sliding contact behaviours under the rotation‑failure condition of TBM central cutter. A series of steel‑needle scratching tests under constant normal load were carried out on dry and water‑saturated weakly cemented sandstone (WCS), aiming at the pure‑sliding contact feature under such failure condition. Featuring low cost and good repeatability, this testing method provides a reliable laboratory approach for investigating the tip‑geometry evolution of central hobs in WCS strata. The main conclusions are drawn as follows:

(1) Test results show that the displacement corresponding to the first sharp drop of mean frictional resistance for water-saturated specimens is 1.8–1.9 times that of dry specimens. For 0.4 mm and 1.0 mm steel needles scratching WCS, the relative tip-diameter variation increases by 46.33% and 49.46% under water-saturated condition compared with dry condition. The observed behavior suggests that clay-mineral cement in WCS softens after water immersion, leading to rock strength reduction and improved ductility. Rock failure tends to transfer from brittle spalling to ductile shear. Increased rock plasticity may promote larger tunnelling-induced deformation and cutting resistance and produce extra frictional energy dissipation, which may aggravate the geometric deterioration of tool tip. The observed behaviours suggest that enhanced rock plasticity and secondary interfacial abrasion may account for the relatively high tunnelling energy consumption of TBM in water-rich weakly cemented strata.
(2) Significant differences are observed between scratching tests using two sizes of cemented‑carbide probes. For the 1.0 mm steel needle, frictional resistance remains stable within 65–75 N with a range no larger than 30 N. By contrast, the frictional resistance of the 0.4 mm steel needle varies from 60 N to 85 N with violent load fluctuation, and the maximum range reaches 42–65 N for different test groups. High stress concentration occurs within the contact zone of small diameter needles, which are more sensitive to the heterogeneous internal structure of WCS. Sustained alternating load may aggravate local geometric deterioration of the needle tip. This experimental observation is comparable to inservice characteristics of field TBM cutters: central hobs with narrow blade width suffer large force fluctuation and prominent stress concentration, showing poorer stability compared with wider‑blade hobs. Tool damages such as eccentric wear and edge chipping are frequently reported in field projects.
(3) Equivalent blade width and rock mass water content are the key sensitive factors governing frictional‑resistance evolution and tip‑geometry variation during scratching. Scratching depth and relative tip‑diameter variation follow a consistent order: 0.4B > 0.4G > 1.0B > 1.0G. Under rotation failure status, central hobs continuously scrape rock in sliding mode. In WCS strata, small‑diameter needles representing narrow‑blade hobs are prone to stress concentration, achieving stronger rock‑cutting capacity. Deeper scratch grooves and newly formed free surfaces can improve rock-breaking performance, yet this condition may aggravate the geometric deterioration of tool edges. The doublecentral hob layout widely adopted in practical TBM projects aims to reduce the peak contact stress on individual hob. It preserves the rock‑breaking advantage of narrow-blade cutters while mitigating edge damage under rotation-failure conditions. Practical engineering experience corroborates the findings obtained from laboratory tests.

It should be pointed out that the scaled steel needle engraving in this article is a quasi-static simplified comparative test, which can only reproduce part of the blade rock interaction mechanism and cannot be fully equivalent to the real service conditions of TBM cutters. When applying the research results to engineering practice, it is necessary to comprehensively judge based on the on-site conditions.

**Author Resume**: Yang Qing is a female PhD, postdoctoral fellow, and lecturer. Her main research interests are geotechnical engineering and underground engineering. email runzey@chzu.edu.cn

## Supporting information

S1 File Supporting data and raw measurements for the scratch test on WCS specimens. The compressed archive contains the following items: (1) model calculation parameters; (2) needle diameter measurements; (3) raw friction-displacement curves; (4) repeated scratch measurements; (5) scanning electron microscope images of WCS at different magnifications; and (6) XRD quantitative analysis data.
(RAR)
